# Automatic classification of experimental models in biomedical literature to support searching for alternative methods to animal experiments

**DOI:** 10.1186/s13326-023-00292-w

**Published:** 2023-09-01

**Authors:** Mariana Neves, Antonina Klippert, Fanny Knöspel, Juliane Rudeck, Ailine Stolz, Zsofia Ban, Markus Becker, Kai Diederich, Barbara Grune, Pia Kahnau, Nils Ohnesorge, Johannes Pucher, Gilbert Schönfelder, Bettina Bert, Daniel Butzke

**Affiliations:** 1 German Centre for the Protection of Laboratory Animals (Bf3R), German Federal Institute for Risk Assessment (BfR), Berlin, Germany; 2 Current affiliation: Nuvisan ICB GmbH, Müllerstraße 178, 13353 Berlin, Germany; 3 Institute of Clinical Pharmacology and Toxicology, Charité - Universitätsmedizin Berlin, Charitéplatz 1, 10117 Berlin, Germany

**Keywords:** Alternatives to animal experiments, Corpus annotation, Text classification, Replacement

## Abstract

Current animal protection laws require replacement of animal experiments with alternative methods, whenever such methods are suitable to reach the intended scientific objective. However, searching for alternative methods in the scientific literature is a time-consuming task that requires careful screening of an enormously large number of experimental biomedical publications. The identification of potentially relevant methods, e.g. organ or cell culture models, or computer simulations, can be supported with text mining tools specifically built for this purpose. Such tools are trained (or fine tuned) on relevant data sets labeled by human experts. We developed the GoldHamster corpus, composed of 1,600 PubMed (Medline) articles (titles and abstracts), in which we manually identified the used experimental model according to a set of eight labels, namely: “in vivo”, “organs”, “primary cells”, “immortal cell lines”, “invertebrates”, “humans”, “in silico” and “other” (models). We recruited 13 annotators with expertise in the biomedical domain and assigned each article to two individuals. Four additional rounds of annotation aimed at improving the quality of the annotations with disagreements in the first round. Furthermore, we conducted various machine learning experiments based on supervised learning to evaluate the corpus for our classification task. We obtained more than 7,000 document-level annotations for the above labels. After the first round of annotation, the inter-annotator agreement (kappa coefficient) varied among labels, and ranged from 0.42 (for “others”) to 0.82 (for “invertebrates”), with an overall score of 0.62. All disagreements were resolved in the subsequent rounds of annotation. The best-performing machine learning experiment used the PubMedBERT pre-trained model with fine-tuning to our corpus, which gained an overall f-score of 0.83. We obtained a corpus with high agreement for all labels, and our evaluation demonstrated that our corpus is suitable for training reliable predictive models for automatic classification of biomedical literature according to the used experimental models. Our SMAFIRA - “Smart feature-based interactive” - search tool (https://smafira.bf3r.de) will employ this classifier for supporting the retrieval of alternative methods to animal experiments. The corpus is available for download (https://doi.org/10.5281/zenodo.7152295), as well as the source code (https://github.com/mariananeves/goldhamster) and the model (https://huggingface.co/SMAFIRA/goldhamster).

**Supplementary Information:**

The online version contains supplementary material available at 10.1186/s13326-023-00292-w.

## Introduction

Current animal welfare legislation, like the Directive 2010/63/EU^1^ from the European Union (EU) and the US Animal Welfare Act^2^, allow to perform an animal experiment addressing a particular research question, only if no alternative method is already available. Therefore, in the process of obtaining approval for an animal experiment, researchers are required to carry out a comprehensive search to ensure that an alternative method is not yet available. This is a time consuming and complex task that involves many queries to databases with references to scientific publications, and careful screening of candidate publications.

There are two important aspects that should be evaluated by researchers when screening for suitable literature: (i) whether the candidate publication’s scientific objective is the same as the one that is planned; and (ii) whether the candidate describes an experimental model other than a living (vertebrate) animal. The later complies with the principle of “replacement”, as part of the 3R principles [1]. These principles provide a framework for the development of alternatives methods that are able to replace an animal experiment, reduce the number of animals, or refine the experiments to increase the welfare of animals. In this work, we focus on the second aspect above, i.e., to support finding experimental models other than living animals. To date, there is no tool that supports the search for alternative methods in the literature.

In order to identify whether an experimental approach described in a publication complies with that aspect, the first step is to identify the type of experimental model that is used. While the EU Directive protects all vertebrate animals (mammals, birds, fish, etc.), it does not protect invertebrates, except cephalopods (e.g. octopuses). Therefore, invertebrate animals represent alternative models, and methods based on such animal models are considered alternative methods. Moreover, most in vitro methods (i.e. cell cultures) comply with the replacement principle, and so do experimental computer simulations (in silico methods). So-called ex vivo methods still rely on animals to provide organs or tissues for subsequent experiments, but no live animal experiment is performed, so such methods are not considered animal experiments for the purposes of Directive 2010/63/EU.

The aim of this work is to develop a computational method for automatic classification of publications with respect to the experimental model. Such a classification is an important part of a tool specialized in finding alternative methods, and it will be included in our corresponding SMAFIRA (SMArt Feature based Interactive RAnking) Web Tool, which is currently being developed in our research group.

Since more than one experimental model can be described in experimental biomedical publications, we tackled the task as a multi-label document classification, i.e., the automatic assigning of one or more labels to a textual document. Our classification scheme consists of eight labels, and a group of 13 individuals with biomedical expertise annotated a set of 1,600 PubMed^3^ articles (titles and abstracts). We chose to rely on PubMed because it is the largest freely available database with references to biomedical publications and it provides Web services for querying and retrieving articles^4^. We experimented with different classifiers to evaluate their ability to classify the experimental models described in the abstracts. Throughout the paper, we mention “articles” or “abstracts” when referring to the titles and abstracts.

In summary, these are the contributions of this publication:

- The annotation of a novel corpus of scientific abstracts in which we manually assigned the used experimental model. This is also a new benchmark corpus for multi-class, multi-label text classification, a task for which few corpora from the biomedical domain are currently available.
- Python scripts for the machine-learning methods that we experimented with using our corpus.

This article has the following structure: Section “Related work” describes related work for the identification of the used experimental model in publications. In Section “GoldHamster corpus”, we describe the development of our corpus, including the selection of the labels, the retrieval of the documents, and the rounds and quality assessment of annotations. We explain the machine learning methods that we employed in our experiments in Section “Methods”. Section “Results” presents the results that we obtained, i.e., statistics of the annotated corpus and the performance of the methods on the corpus. Finally, we discuss some interesting aspects of our corpus and experiments in Section “Discussion”.

## Related work

Some previous attempts aimed to identify alternative methods (or 3R-relevant methods) in PubMed. Among others, they have relied on curated lists of relevant MeSH (Medical Subject Headings) terms, for instance, to identify publications containing a method based on a cell culture model. ALTBIB^5^, i.e. the “bibliography on alternatives to the use of live vertebrates in biomedical research and teaching”, uses this approach. ALTBIB automatically adds MeSH terms to the user query, e.g., the terms “in silico” and “QSAR” when searching for in silico methods. However, such predefined search strategies quickly become obsolete as new and potentially relevant MeSH-terms are continuously added to PubMed.

In addition to the various MeSH terms that can be used to identify certain classes of experimental methods, there are two terms specially designed to retrieve alternatives to animal experiments as a whole, namely, “Animal Testing Alternatives” and “Animal Use Alternatives”. We have elaborated some case studies of alternatives to animal experiments in the domains of Parkinson’s disease, Huntington’s disease, breast cancer and stroke before [2] and did not identify the above mentioned specific MeSH-terms in any of the relevant publications. Thus, relevant experimental models and methods may simply be missed when only relying on these two MeSH terms.

A recent work describes the development of a corpus and method for predicting alternatives to animal experiments, or 3R-based literature, based on these two MeSH terms [3]. The authors initially collected around 4,000 citations from PubMed associated with these terms, which was compared to a random set of citations of the same size. They relied on the word2vec algorithm to reveal meaningful patterns, which are then used in a random forest model. However, more details about this research is not yet available, and neither are the data nor the methods.

To the best of our knowledge, there is no manually annotated corpus that can be used for supervised machine learning and the automatic classification of biomedical publications according to used experimental model. However, there are databases that can help with the identification of alternatives to animal experiments. For instance, the Non Animal Technologies (NAT) database^6^ provides a collection of non-animal technologies that is available for download from their Web site. The current state of the database comprises 1,780 entries (as of June/2023) and it includes the identification of the type of method or model, e.g. “human studies”, “epidemiology”, “in silico”, “artificial intelligence”. Such databases, however, are very limited in their coverage, since collection of data essentially requires human efforts. Moreover, not all entries have a link to a publication, and the database is not transparent about the selection of the proposed methods and who is responsible for this procedure. Further, we are also aware of the 3Ranker^7^, which provides a list of curated alternatives to animal experiments. As of June/2023, the tool contains 121 curated publications.

Another important collection of non-animal models is the reports that the EU Commission regularly releases for some specific topics. As of June/2023, we are aware of seven reports for the areas of breast cancer [4], respiratory tract diseases [5], neurodegenerative diseases [6], immuno-oncology [7], immunogenicity testing for advanced therapy medicinal products [8], cardiovascular diseases [9], and autoimmune diseases [10].

The corresponding data for each report can be downloaded as a spreadsheet, and similar to the NAT database, it includes information about the methods or models. The collected models, however, are predominantly human-based and thus miss a great portion of candidate alternative models that based upon animal tissues and cells. In addition, these reports describe advanced non-animal methodologies that do not necessarily replace existing animal-based methods.

The annotations in our corpus overlap with some previous corpora that addressed named-entity recognition (NER), for instance, of species [11, 12], anatomical parts [13], or cell lines [14]. State-of-the-art methods for NER are mostly based on the transformers architecture and a pre-trained model, e.g., BioBERT [15] or PubMedBERT [16], and a fine-tuning on NER-specific corpora. Further, some NER tools are readily available for use, such as PubTator [17] and BERN2 [18]. However, as opposed to usual NER tasks, we do not aim to identify all mentions of these entities, but focus only on cases that occur in the context of the used model.

Furthermore, not all mentions of vertebrate animals necessarily correspond to an in vivo experiment, i.e. using living animals. If the animal was killed in advance for the removal of organs, tissues, or some cells, such an approach is not considered an animal experiment according to the Directive 2010/63/EU. Within the classification schema that we propose, the depicted situation alludes to different labels: “organs” and/or “primary cells”. Finally, we are not aware of previously developed NER tools for the extraction of in silico methods, which is one of the labels that we consider.

Comprehensive ontologies and thesauri for the biomedical domain, such as the MeSH terms, address a wide range of concepts related to some of the labels that we consider. For example, there are MeSH terms for “primary cell culture” or “cell line”. Anyway, these terms do not differentiate between the source of biological materials. The respective labels used in the first version of our corpus designate materials from vertebrate animals only. Other biological materials, used in experimental biomedical research, are labeled as “human” or “invertebrate”, depending on the source. The basic intention behind our labeling is to allow differentiation between sources and to make a clear distinction between experiments using living animals (“in vivo”) and experiments using materials from animals (“ex vivo”). The MeSH-term “animals” does not allow for such a clear distinction. Therefore, we cannot make use of MeSH terms as a straightforward approach to identify the used experimental model. Indeed, a preliminary analysis of the correlation of our labels with the respective MeSH terms concluded that a simple mapping between them is not feasible (cf. Section “Correlation with the initial database queries and the corresponding MeSH terms”).

We are only aware of one ontology which was built specifically for the 3R domain [19], namely the one developed to support the Go3R search engine [20]. Go3R was one of the first attempts to develop a tool for finding alternative methods to animal experiments. Its ontology was divided in 28 branches and contained more than 16,000 concepts. The tool utilized a maximum entropy algorithm, which was trained on a collection of 3,000 manually annotated documents, to predict whether an article was 3R-relevant. However, the tool is no longer available, and the methods, the training data, and the ontology were never released.

## GoldHamster corpus

We annotated 1,600 PubMed abstracts to support classification according to the experimental models they use. In this section, we describe: (a) how we defined the annotation scheme, (b) the queries we used to search for abstracts, and (c) details of the annotation process.

### Definition of the annotation schema

In order to display the 3R strategies (replace, reduce, refine), we initially thought of three groups representing the degree of invasiveness of an experiment: (i) animal experiments that take place in a living organism (so called *in vivo* experiments in scientific articles); (ii) experiments taking place in isolated organs/tissues explants or primary cells from organisms sacrificed in advance; and (iii) research conducted with no need of using any laboratory animal or material from animals. However, the usefulness of these three categories for researchers is limited. Therefore, we created eight specific labels indicating not only the level of invasiveness but also the species or the origin of material used (see overview in Table 1).

In particular, the label “human” covers all experiments conducted in human/patients and or in any kind of human material (including organs/tissues, primary cells and immortal cells). The label “invertebrate” refers to all experiments using invertebrates or invertebrate material (mainly flies and “worms”) - excluding cephalopods. As a tribute to the Directive 2010/63/EU that protects cephalopods in the same way like vertebrates, our schema places experiments with cephalopods together with vertebrates. In addition, research related to vertebrates and cephalopods was subdivided further in the following labels: (a) “in vivo” for live animals, (b) “organs” for isolated organs/tissues, (c) “primary cells” for freshly isolated primary or stem cells, and (d) “immortal cell lines” for established cancer or immortal cell lines that can be ordered from cell and tissue collections, e.g., the American type culture collection (ATCC). The label “in silico” was included to indicate research using computer simulations. We labeled any observational (but not experimental) study as “other”, e.g. clinical retrospective studies.

**Table 1:** List of labels in the GoldHamster corpus. The annotators could assign one or more label to an abstract, or even no label at all. *excluding cephalopods **including cephalopodes

Label	Description	Examples
human	humans/material	“patients received a bolus injection of”
invertebrate	invertebrates/material*	“drosophila (se) were exposed to”
in vivo	living vertebrates**	“blood pressure telemetry in rat”
organs	vertebrate organs/tissues	“mouse isolated perfused kidney”
primary cells	vertebrate primary/stem cells	“Murine neural stem cells were isolated”
immortal cell lines	vertebrate immortal cell lines	“Hela, CHO, MDCK”
in silico	computer simulations	“digital simulation of food digestion”
others	any other kind of experiments	“retrospective studies, meta-analysis”

### Retrieval of abstracts

Complex search strategies using MeSH (Medical Subject Headings) were devised to retrieve the abstracts and searches were performed in PubMed/MEDLINE (on August 20, 2019). The queries consisted of combinations of MeSH-terms referring to certain experimental models and techniques (e.g. “Animals, Genetically Modified[MeSH] OR Animal Experimentation[MeSH] OR ...”) and relevant categories (e.g. “Diseases Category[MeSH]”) with filters (e.g. “English[lang]”). Such combinations then were extended to target certain clusters of MEDLINE-abstracts. The terms which were searched for cluster headings were “in vivo”, “organs and tissues”, “primary and stem cells”, “immortalized and tumor cells”, “in silico”, “invertebrates”, “humans”, “other” (cf. Section 1 in the supplementary material). From every retrieved list, the first 200 abstracts were downloaded and were included in the corpus. We created eight queries, i.e., one for each cluster, and they yielded a combined corpus of 1,600 abstracts. We provide our queries in the supplementary material (Section 1).

### Annotation process

A group of 13 annotators carried out the annotation using the TeamTat tool^8^ [21]. All annotators have a doctoral degree in the field of biomedical science or are currently research assistants in our department. The annotation guideline is provided in the supplementary material (Section 2). The annotation process was carried out in two rounds: (i) round 1, namely “r1”, in which each of the 1,600 abstracts was annotated by two annotators, who were randomly selected; (ii) round 2, which was split into four short rounds, namely “r2.1”, “r2.2”, “r2.3”, and “r2.4”, in which a selected team of the annotators resolved the disagreements in annotations from the first round. We describe both rounds in details below.

*First round - r1*. We arranged the 1,600 abstracts in 40 units of 40 abstracts each and assigned them to annotators. Each annotator received a set of four (i.e., 160 abstracts) to eight (i.e., 320 abstracts) units. The annotators were required to highlight the applied experimental models, which are described in either the title or text of the abstract. Even though we addressed the problem as a text classification task, highlighting a text span was necessary because TeamTat does not support document-level annotation. However, for the sake of simplicity, we asked the annotators to highlight only one mention (text span) for each label, instead of all mentions of the experimental model in the text. In addition, we did not specify which text should be highlighted, since only the labels were relevant. The annotators were encouraged to consult external resources in the Web, such as Cellosaurus^9^ [22] or ATCC^10^, for the identification of the origin of a cell line. Finally, if the abstract did not allude to any experimental model, the annotators were asked not to assign a label to it.

*Second rounds - r2.1, r2.2, r2.3, r2.4.* In the second rounds, five selected annotators^11^ resolved the disagreements in annotations from the first round. In this round, we aimed to achieve an agreement for the annotations, by requiring two annotations from distinct annotators for the labels. The selected annotators had to consider which of the two (anonymous) annotators in the first round was correct. If the annotator conceived a third opinion about the labels, the abstract was flagged to be removed, since no agreement between any two annotators was obtained. This phase consisted of four short rounds. For the three first rounds, we selected some particular disagreement combinations of labels from the first round. All rounds are listed below:

- r2.1: 71 abstracts in which one annotator assigned only the label “invertebrate”, while the other one assigned something else, i.e. one label other than “invertebrate” or multiple labels (which could also include “invertebrate”);
- r2.2: 114 abstracts in which one annotator assigned only the label “human”, while the other one assigned something else, i.e. one label other than “human” or multiple labels (which could also include “human”);
- r2.3: 148 abstracts in which one annotator assigned only the label “in vivo”, while the other one assigned something else, i.e. one label other than “in vivo” or multiple labels (which could also include “in vivo”).
- r2.4: 368 abstracts that remained.

## Methods

In this section, we describe the classifiers (and additional semantic features) that we trained to automatically assign labels to abstracts.

### Classifiers

We experimented with the current state-of-the-art approach for the biomedical natural language processing (BioNLP) domain, i.e. fine-tuning a pre-trained Bidirectional Encoder Representations from Transformers (BERT) model [23] on our annotated corpus. We considered only models which were either specific for the biomedical domain or for the scientific literature. These pre-trained language models achieved state-of-the-art results for some BioNLP tasks (e.g. text classification, question answering, and named entity recognition) without requiring substantial modification in the model architecture [16].

We relied on the implementation of BERT in the Transformers model^12^ for TensorFlow. In our experiments, we used the Adam optimizer, epsilon of $1 \times 10^{-8}$ and a decay of 0.01. We experimented with various values to adjust the model hyperparameters, such as learning rate, batch size, and the number of epochs, as later described in Section “Corpus evaluation”. The language models that we considered were the following: (a) BioBERT^13^ [15], (b) PubMedBERT^14^ [16], (c) SciBERT^15^ [24], and (d) BLUE-BERT^16^ [25].

### Additional semantic features

In additional to the title and text of the abstracts, we also considered variations of the text of the abstracts that was used as input to the classifiers. The text was either expanded with the addition of the corresponding MeSH terms, or reduced by considering only some particular parts of it. We describe these two approaches below.

#### MeSH terms

We assessed whether the addition of the MeSH terms to the text of the abstracts could boost the performance of the results. Our assumption is that these terms reflect the important content of articles. However, some of these terms might refer to information only present in the full text of articles, while our annotation was based only on the abstracts.

We considered all MeSH terms originally assigned to the articles, as well as subsets of these based on pre-defined thresholds, i.e., we considered only the ones that occurred in at least a certain number of articles. There were 5,158 distinct MeSH terms in our corpus of 1,600 articles. When experimenting the values of n = 10, 25, 50, and 100 for the threshold, i.e. occurrence of MeSH terms in at least “n” articles, the number of distinct terms was reduced to 298, 93, 38, and 15, respectively. In the supplementary material (Section 3), we depict the variation in the number of terms per article for each of these thresholds. In our experiments, we concatenated the terms at the beginning of the text, in addition to the title and abstract text.

#### Discourse elements (sections)

We evaluated whether concentrating on only potentially relevant parts (sections) of the abstracts, e.g., background or methods, could improve the performance of the classifier. As opposed to extending the text of the articles (cf. MeSH terms above), we reduced the text by considering only some sections of the abstracts. For this purpose, we relied either on the original sections in PubMed or the ones predicted by the ArguminSci tool [26], which was the best performing tool for this task in our previous evaluation [27].

We first checked the sections as provided by PubMed in the structured abstracts. Out of our 1,600 articles, only 464 of them were structured. From these structured abstracts, we obtained 81 distinct section names. We considered only the ones that appeared in least in ten structured abstracts, and which referred to the usual sections in scientific publications. We provide the list of these sections in the supplementary material (Section 4). For the abstracts that were not structured, we considered the sections as automatically predicted by the ArguminSci tool^17^. We restricted to a set of five labels, namely: “Background”, “Objective”, “Methods” “Results”, and “Conclusions”. We mapped these labels to the ones in the structured abstracts and from the ArguminSci tool (cf. Section 4 in supplementary material).

In our approach, we first considered the sections in the structured abstracts, and if not available, we used the ones predicted by ArguminSci. We first experimented with each of the sections, i.e., the experiments utilized only the text that was included in one particular section. Subsequently, we studied a combination of the sections which obtained a good performance in the first round of experiments. For all experiments, we build the text by concatenating the selected section(s) in the original order that they appear in the abstract. We did not consider the title in any of the experiments.

## Results

In this section, we present an analysis of the manual annotations that we obtained for the GoldHamster corpus, and the results of the experiments with automatic prediction of the labels.

### Corpus analysis

After the first round of annotation, in which two annotators screened each of the articles, we obtained 7,737 annotations^18^: 1,970 for “in vivo”, 1,397 for “human”, 1,171 for “invertebrates”, 892 for “others”, 740 for “organs”, 663 for “in silico”, 455 for “primary cell lines”, and 449 for “immortal cell lines”. These values are the total of annotation with duplicates, i.e. including situations in which two annotators agreed in assigning a certain label to an abstract. In addition, in 190 cases, an annotator did not assign any of the labels to an abstract. From the 1,600 abstracts, 899 had a full agreement, i.e. exactly the same sets of labels were assigned by both annotators. The remaining 701 abstracts without full agreement were reviewed in the additional rounds (r2.1, r2.2, r2.3, and r2.4).

**Table 2:** Statistics of the corpus in terms of the number of abstracts per label. We present statistics for all labels (cf. 3.1), rounds of annotation (cf. 3.3) and when considering all annotations (All, left side) or only the one for which two annotators agree (Agree, right side). The comparison for the rounds in terms of equality (=), increase ($\bigtriangleup$), or decrease ($\bigtriangledown$) is with respect to the previous column (round), i.e. after the addition of the corresponding round. The values do not include duplicates, i.e. a label is only counted once if there is an agreement for it

Labels	r1	+ r2.1	+ r2.2	+ r2.3	+ r2.4
	All/Agree	All/Agree	All/Agree	All/Agree	All/Agree
invertebrates	263/191	14$\bigtriangledown$/15$\bigtriangleup$	=/=	2$\bigtriangledown$/=	6$\bigtriangledown$/6$\bigtriangleup$
in_vivo	483/338	1$\bigtriangledown$/1$\bigtriangleup$	=/1$\bigtriangleup$	19$\bigtriangledown$/19$\bigtriangleup$	7$\bigtriangledown$/50$\bigtriangleup$
in_silico	216/124	4$\bigtriangledown$/3$\bigtriangleup$	2$\bigtriangledown$/3$\bigtriangleup$	1$\bigtriangledown$/7$\bigtriangleup$	28$\bigtriangledown$/20$\bigtriangleup$
human	325/165	1$\bigtriangleup$/=	37$\bigtriangledown$/37$\bigtriangleup$	4$\bigtriangledown$/2$\bigtriangleup$	23$\bigtriangledown$/20$\bigtriangleup$
organs	261/128	3$\bigtriangledown$/=	=/=	47$\bigtriangledown$/11$\bigtriangleup$	28$\bigtriangledown$/22$\bigtriangleup$
immortal_cell_lines	160/55	2$\bigtriangledown$/=	23$\bigtriangledown$/5$\bigtriangleup$	2$\bigtriangledown$/6$\bigtriangleup$	5$\bigtriangledown$/38$\bigtriangleup$
primary_cells	189/59	6$\bigtriangledown$/=	1$\bigtriangledown$/=	22$\bigtriangledown$/6$\bigtriangleup$	37$\bigtriangledown$/29$\bigtriangleup$
others	402/130	23$\bigtriangledown$/5$\bigtriangleup$	25$\bigtriangledown$/35$\bigtriangleup$	18$\bigtriangledown$/7$\bigtriangleup$	44$\bigtriangledown$/57$\bigtriangleup$
none	165/25	11$\bigtriangledown$/=	=/=	=/=	89$\bigtriangledown$/=
total docs	1,600/1,168	=/21$\bigtriangleup$	=/68$\bigtriangleup$	=/32$\bigtriangleup$	=/147$\bigtriangleup$

We summarize the number of abstracts for label that we obtained after each of the annotations rounds in Table 2. We show the impact of each additional round as compared to the previous column. Furthermore, we present results when considering all annotations (“All”) and when only considering the ones for which there was an agreement between two annotators (“Agree”). As expected, when full agreement is considered, the number of articles with any annotation reduces, i.e., from 1,600 to 1,168 for the first round. While only 899 abstracts had a full agreement, 1,168 abstracts had an agreement for at least one of the labels. Further, the number of abstracts with agreement increases after the annotation of the additional rounds, namely to: 1,189 (after r2.1), 1,257 (after r2.2), 1,289 (after r2.3), and 1,436 (after r2.4). For all rounds, the number of documents without any annotation remained equal to 25.

In the four additional rounds, the selected annotators received the anonymous annotations from the two annotators in the first round and were required to decide which annotation is the correct one. If no first round annotation was judged correct, the respective abstracts were removed from the corpus. We removed the following number of abstracts from the additional rounds: 11 (from 71) in r2.1, 20 (from 114) in r2.2, 23 (from 148) in r2.3, and 77 (from 368) in r2.4. We present the list of PMIDs in the supplementary material (Section 5).

**Table 3:** Agreement in terms of Cohen’s ($\kappa$) between annotators for the first round, for each label (cf. 3.1), and for the overall corpus. An agreement is moderate if the ($\kappa$) is higher than 0.6, and strong if higher than 0.8 (cf. [29])

Labels	Kappa	Level Of agreement
invertebrates	0.82	almost perfect
in vivo	0.78	substantial
in silico	0.72	substantial
human	0.63	substantial
organs	0.62	substantial
immortal cell lines	0.49	moderate
primary cell lines	0.45	moderate
others	0.42	moderate
overall	0.62	substantial

In Table 3 we show the level of agreement between the annotators with respect to individual labels in terms of the kappa coefficient ($\kappa$) [28]. Regarding the first round, we obtained an almost perfect agreement for “invertebrates” (0.82), substantial agreement for “in vivo” (0.78), “in silico” (0.72), “human” (0.63), and “organs” (0.62), and moderate agreement for “immortal cell lines” (0.49), “primary cells” (0.45), and “others” (0.42). Two of the lowest agreements were for the “primary cell lines” and “immortal cell lines” labels, which are indeed difficult to distinguish (if not using Cellosaurus).

### Corpus evaluation

We ran various experiments to evaluate the corpus for predicting the labels. All results are in terms of the standard metrics of precision, recall, and f-score. In order to perform a 10-fold cross validation, we split the collection of abstracts into 10 parts in a stratified way, i.e., in a way to obtain datasets with a similar distribution of labels as in the complete corpus (cf. corpus statistics in Section 6 of the supplementary material).

The hyperparameters that we considered were decided based on an evaluation with a preliminary version of our corpus (after round 2.3) and with BioBERT. The sets of values for the hyperparameters that we considered were the following: learning rate of $1 \times 10^{-5}, 5 \times 10^{-5}, 1 \times 10^{-4}$, and $5 \times 10^{-4}$; batch size of 16 and 32; and epochs of 10, 20, 30, 40, and 50. We show the results for the 40 experiments that we ran with all combinations of hyperparameters in the supplementary material (Section 7). We made the final decision of the hyperparameters based on this analysis and on some of our constraints, i.e., time and memory performance. For our further experiments, we used the following hyperparameters: maximum length of 256, learning rate of $1 \times 10^{-4}$, batch size of 32, and 10 epochs.

We compared the various language models based on our best run in the 10-fold cross validation. Table 4 summarizes the results. Further, we compared our best performing model to the various SVM kernel functions (cf. Section 8 the in supplementary material).

**Table 4:** Performance (in f-score) for the various pre-trained language models

Labels	BioBERT	PubMedBERT	SciBERT	BLUE-BERT
invertebrates	0.89	**0.95**	**0.95**	**0.95**
in_vivo	0.88	0.88	**0.89**	0.83
human	0.82	**0.86**	0.82	0.73
organs	0.80	**0.82**	**0.82**	0.71
primary_cells	0.67	**0.75**	0.73	0.67
immortal_cell_lines	**0.91**	0.83	0.80	0.89
in_silico	0.67	0.75	0.75	**0.86**
others	0.70	**0.78**	0.67	0.76
All (average)	0.79	**0.83**	0.80	0.80

In average, PubMedBERT performed slightly better than the other language models. It provided scores of at least 0.75 for all labels, while all other models provided a score under 0.70 for one or more labels. Indeed, PubMedBERT obtained a very good performance for other text classification tasks, as listed in the BLURB leaderboard^19^.

### Evaluation with the addition of MeSH terms

For the best performing model that we obtained with PubMedBERT (cf. Table 4), we ran experiments for all the proposed threshold values (cf. Section “MeSH terms”). Table 5 summarizes the results. Only one threshold obtained a performance as high as the experiment without the use of MeSH terms, namely, the value of 10 (overall f-score of 0.83). However, for all labels, there was always a threshold value that outperformed the experiment without the MeSH terms.

**Table 5:** Prediction of the labels with or without (w/o) adding the MeSH terms. We considered various thresholds for the minimum frequency of the terms, in relation to the number of articles in which they appear

	w/o MeSH	thresholds				
		0	10	25	50	100
invertebrates	0.95	**1.00**	**1.00**	0.89	0.89	0.90
in_vivo	0.88	0.88	0.88	**0.89**	**0.89**	0.88
human	0.86	0.83	0.76	0.87	**0.91**	0.70
organs	0.82	**0.84**	0.82	0.75	0.82	**0.84**
primary_cells	0.75	0.60	0.67	**0.89**	0.67	0.80
immortal_cell_lines	0.83	0.91	**1.00**	0.77	0.75	0.71
in_silico	0.75	**0.87**	0.80	0.80	0.80	0.75
others	0.78	0.64	0.69	0.72	**0.80**	0.59
All (average)	**0.83**	0.82	**0.83**	0.82	0.82	0.77

### Evaluation with the addition of discourse elements

For the best performing model that we obtained with PubMedBERT (cf. Table 4), we ran experiments with each of the sections as well as some of their combinations. Table 6 summarizes the results. None of these experiments outperformed the one that considered the titles and abstracts. Some sections achieved a better performance than others, namely, “Background”, “Methods” and “Conclusions”. Therefore, we ran experiments only for combinations of these three sections.

**Table 6:** Performance (in f-score) when using individual and combined sections for the prediction of the abstract labels. We show results from considering each section separately, i.e. Background (B), Objective (O), Methods (M), Results (R), and Conclusions (C), as well as combinations of the three best performing ones

	none	B	O	M	R	C	BM	BC	MC	BMC
invertebrates	**0.95**	**0.95**	0.76	0.89	0.95	0.95	1.00	0.84	0.82	**0.95**
in_vivo	**0.88**	0.76	0.75	0.86	0.83	0.79	0.83	0.70	0.80	0.83
human	0.86	**0.90**	0.62	0.78	0.67	0.70	0.78	0.78	0.73	0.80
organs	**0.82**	0.71	0.53	0.75	0.63	0.67	0.74	0.59	0.53	0.75
primary_cells	0.75	0.40	0.44	0.50	0.29	0.67	0.57	0.25	0.00	**0.86**
immortal_cell_lines	0.83	**1.00**	0.50	0.40	0.36	0.91	0.83	0.75	0.44	0.91
in_silico	0.75	0.67	0.67	0.60	0.80	0.67	0.75	0.80	**0.83**	0.67
others	**0.78**	0.73	0.56	0.48	0.40	0.64	0.64	0.73	0.67	0.70
All (average)	**0.83**	0.76	0.60	0.66	0.62	0.75	0.77	0.68	0.60	0.81

### Comparison to available NER tools

We compared our results to two available tools for NER and entity normalization, namely, BERN2 [18] and PubTator [17]. Both tools extract a variety of entity types, but we considered only predictions for species and cell types, i.e., types “species” and “cell_line” in BERN2, and “Species” and “CellLine” in PubTator. The remaining entity types, e.g., DNA, drugs, diseases, mutations, were not relevant for our schema. Both tools provide the identifiers for the selected types, namely, to the NCBI taxonomy [30] for species and to Cellosaurus [22] for cell lines.

We used the same strategy for both tools. The “human” label was assigned if any prediction linked to *H. sapiens* (NCBITaxon:9606) was found. The abstracts with predictions for either *D. melanogaster* (NCBITaxon:7227) or *C. elegans* (NCBITaxon:6239) were assigned to the “invertebrate” label. For all remaining species, we assigned the abstracts to “in vivo”. Unfortunately, not all predictions for cell lines are mapped to an identifier in Cellosaurus. For the ones for which an identifier was available, we retrieved the corresponding species in Cellosaurus. Similarly, abstracts with cell lines from *H. sapiens* were assigned to the “human” label, and cell lines from *D. melanogaster* to the “invertebrate” label. For all remaining species, we assigned the abstracts to the “immortal cell lines” label, i.e., cell lines from vertebrates. We present results for the prediction of four of our labels in Table 7.

**Table 7:** Prediction of the labels based on the named-entity recognition provided by BERN2 and PubTator. The scores are an average over the 10-fold cross-validation

Labels	BERN2	PubTator
invertebrates	0.54	0.72
in vivo	0.56	0.61
human	0.52	0.45
immortal cell lines	0.24	0.00

For all labels, the score were lower than the ones that we obtained with PubMedBERT. Both NER tools obtained similar results for the “in vivo” and “human” labels. However, BERN2 got some true positives for “immortal cell lines” (as opposed to none from PubTator), while PubTator scored much higher for “invertebrates”. When comparing the number of mentions predicted by each tool, in average, BERN2 predicted a total of 127 cell lines for the test set, as opposed to less than three from PubTator. This probably explain why BERN2 performed better for this label. Actually, BERN2 scored 1.0 for recall, while precision was low, around 0.15 (results not shown).

For the predictions of species, both tools returned approximately the same number of mentions. We checked a couple of abstracts to investigate the source of error, namely PMIDs 26845534 and 28916802. BERN2 correctly detected the “Drosophila” mention, and assigned the type (species). However, no identifier was associated to it, but simply the text “CUI-less”, thus hindering a mapping to the “invertebrate” label.

We also evaluated the two NER tools when considering only annotations that occurred in some particular discourse elements (cf. Section 9 in the supplementary material). We observed only a very small improvement when considering only the background section: for the “invertebrates” and “immortal cell lines” labels with BERN2 and for the “human” with PubTator.

### Sentence-level prediction

We annotated the corpus on the level of the entity span, since this was necessary when working with the TeamTat tool. However, we did not give clear instructions to the annotators on how to annotate the text spans. Further, we did not ask them to annotate all mentions of the particular method, e..g, the species name. Finally, the annotators did not highlight mentions of other methods, if it was not the proposed experimental method. Therefore, our corpus cannot be compared to corpora built for NER task. Nevertheless, we experimented with using the annotations for training a sentence-level model.

The training data consisted of sentences and the corresponding labels of their annotations. We utilized the same code used for training BioBERT on document level, and the only change of parameter was considering the maximum length of 128 (instead of 256), a learning rate of $5 \times 10^{-5}$ (instead of $1 \times 10^{-4}$), and a batch size of 16 (instead of 32). We did not consider a sequence model, as it is usual for NER tasks, i.e., the prediction made for the previous sentence does not influence the prediction for the current sentence. Table 8 summarizes the results. In general, the performance was much higher for the document-level prediction. Curiously, the sentence-level prediction scored better for the “in silico” label.

**Table 8:** Performance (in f-score) for the prediction of the labels on document and sentence level

Labels	documents	sentences
invertebrates	0.91	0.48
in_vivo	0.82	0.41
human	0.74	0.60
organs	0.76	0.33
primary_cells	0.57	0.58
immortal_cell_lines	1.00	0.88
in_silico	0.80	0.89
others	0.73	0.55
All (average)	0.79	0.59

## Discussion

Here we discuss some issues related to our annotations and experiments, such as possible reasons for the inconsistencies in the annotation process, and the correlation of our initial queries to the annotations that we obtained.

### Annotation process

After the first round of annotations, in which 13 annotators independently reviewed 1,600 abstracts with two-fold overlap (to determine inter-annotator agreement), we performed four additional rounds with subsets of first round annotations to partly resolve disagreements from the first round.

As expected, agreement between annotators decreased with the assignment of more than one label per article. For articles where the annotator assigned only one label, we found a full agreement of 71%. This value decreased to 36% when considering two labels per article, and to 12.5% when considering three labels per article.

We identified the labels (and their combinations) for which an inter-annotator agreement was obtained. Table 9 presents the number of articles with such agreement after the first round of annotation. As expected, full agreement occurred mostly in abstracts assigned to just one label. Furthermore, full agreement of annotations was found in 31 abstracts labeled twice, and only six times when three labels were present.

**Table 9:** Number of the 899 articles with full agreement according to the obtained labels. The table is divided into three groups: documents with one, two, or three labels. “Total” is the total number of documents in each group, while “No. Docs” is the number of documents with each of the labels (or set of labels)

	Total	No. Docs	Labels
1 label	862	213	“in vivo”
		156	“invertebrates”
		136	“human”
		97	“others”
		92	“organs”
		85	“in silico”
		30	“immortal cell lines”
		28	“primary cell lines”
		25	none
2 labels	31	6	“in silico+in vivo”
		5	“in vivo+primary cell lines”
		4	“in silico+invertebrates”, or “immortal cell lines+in vivo”, or “in vivo+organs”
		2	“organs+primary cell lines”, or “in silico+primary cell lines”, or “human+invertebrates”, or “in silico+others”
3 labels	6	1	“human+in vivo+primary cell lines”, or “in vivo+invertebrates”, or “immortal cell lines+primary cell lines”, or “in vivo+others”, or “in silico+organs”, or “immortal cell lines+organs”

For abstracts without full agreement, we obtained 167 combinations of disagreements. The most frequent disagreement that we observed was the assignment of the label “human” by one annotator, and the label “others” by the other one (n = 55). Indeed, many of the abstracts assigned to “others” were retrospective studies in which patients were involved. Thus, according to our guideline, the label “others” was the correct one. We provide more details about the disagreements in Section 10 of the supplementary material.

### Correlation with the initial database queries and the corresponding MeSH terms

Our initial assumption was that a query based on MeSH terms could not precisely distinguish between certain types of experimental models^20^, since such terms are too indistinct for this specific task, i.e. to distinguish “animal experiments” from “experiments using animals” (cf. Section “Related work”).

The list of 1,600 PMIDs in our corpus results from eight database queries, which were designed to roughly represent the labels in our schema. From each of the hit lists, we retrieved the top 200 PMIDs for which an abstract was available. After the annotation of the abstracts, we assessed whether the assigned labels in our corpus correspond to our initial queries.

**Fig. 1:**
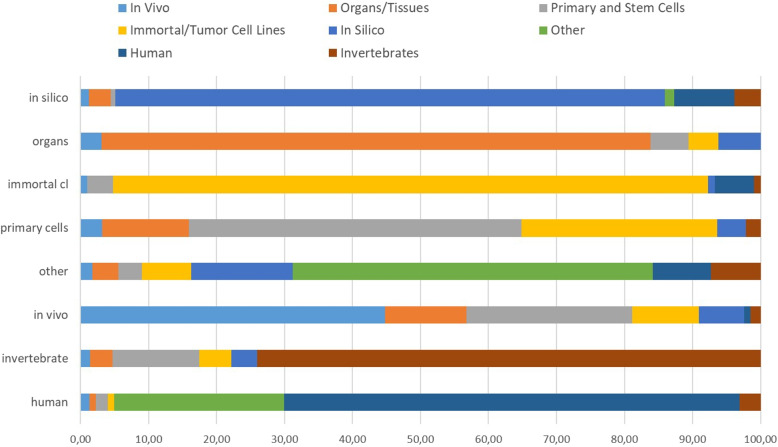
Correlation of the labels (in the graph) with the queries (in the legend) used for retrieving the abstracts. The values represent the percentage of articles assigned to the particular label that correlated with the query. The labels were derived from the annotations after round 2.4

Figure 1 presents the percentage of abstracts that agree with the respective label that was assigned after round 2.4 (i.e. full agreement). The “immortal cell lines”, “organs” and “in silico” labels obtained the highest agreement (over 80%). Furthermore, these values are roughly as high as the f-scores obtained for these labels in our PubMedBERT experiments: 0.83, 0.82 and 0.75, respectively. The correlation for “invertebrate” was high (74.6%), but lower than the f-score for this label, which was of 0.95. The lowest agreement was the one for “in vivo”: only 44.74%.

**Fig. 2:**
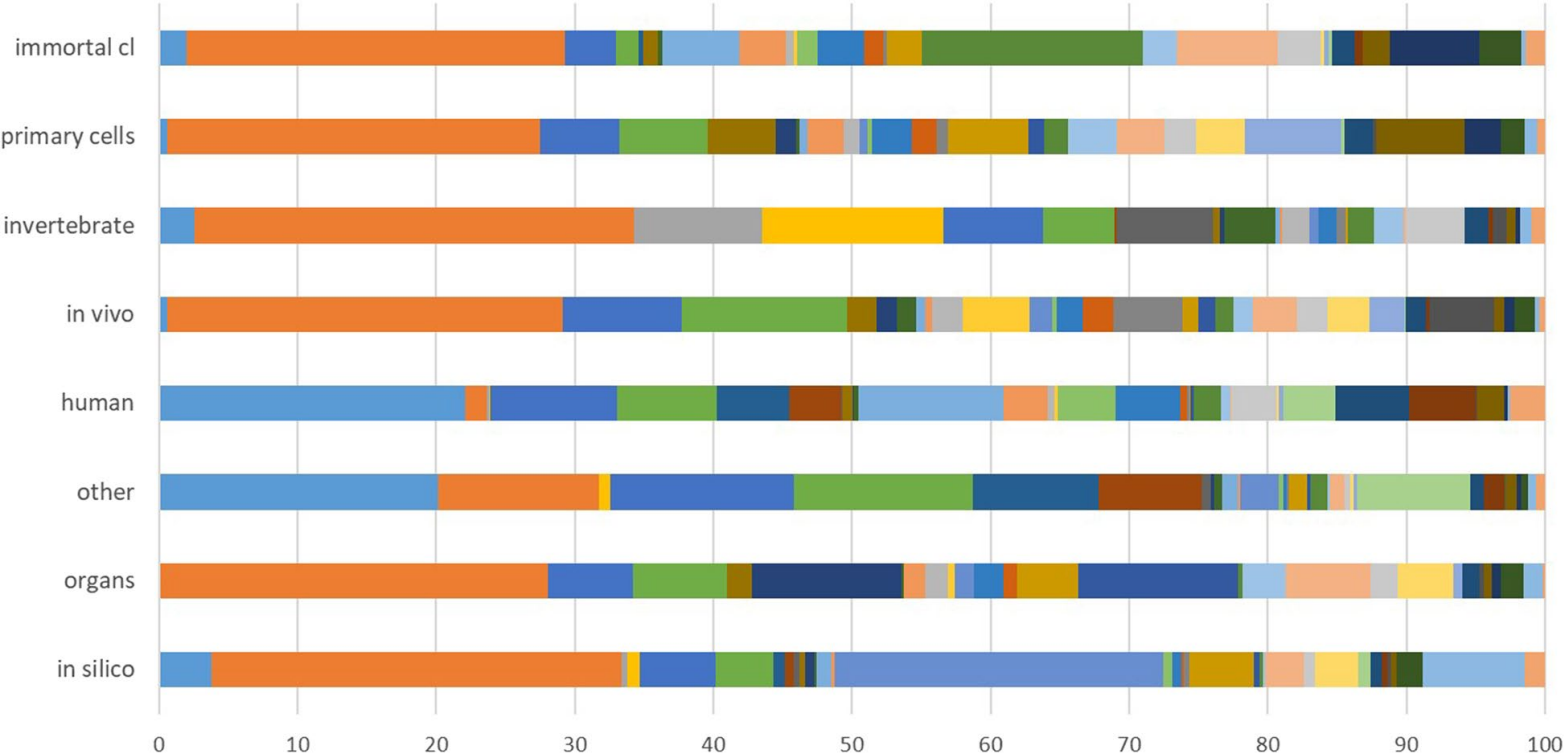
Association of the labels and the top ranked MeSH terms. A variety of colors, i.e., MeSH terms, is associated to all the labels

Further, we assessed the potential of relying on MeSH terms to support the prediction of the labels. We retrieved all MeSH terms associated with our 1,600 abstracts and obtained 2,828 distinct terms when considering only the ones associated to at least ten documents. Only 22 of the abstracts contained no MeSH terms at all. Then we analyzed the correlation between the terms and the labels by checking the MeSH terms that occur in abstracts with a particular label. We summarize in Figure 2 the correlation for the MeSH terms^21^ to a certain label.

On the one hand, some terms had a clear correlation with only one of our labels and are good discriminators, e.g.:

- “Computer Simulation” (large blue element in the middle) for “in silico”;
- “Cell Line, Tumor” (larger light blue element in the middle) for “human”;
- “Drosophila melanogaster” (larger yellow element in the middle) for “invertebrates”;
- and “Rats” (larger dark yellow element in the middle) and “Mice” for “in vivo”.

On the other hand, some terms frequently occur for more than one label, and are not good discriminators, namely the four first elements from the left: “Humans” (light blue), “Animals” (orange), “Female” (dark blue), and “Male” (light green). Therefore, building reliable queries based on MeSH terms is a challenge for most of the labels that we address.

### Limitations and future work

For this first version of our corpus, we designed a set of eight labels. While these labels cover most important vertebrate models in experimental biomedical research, the resolution of human models is underdeveloped. Therefore, we plan to expand the set of labels with classes referring to human models, i.e. “human in vivo”, “human organs/tissues”, “human cell lines”. Furthermore, we plan to improve our guidelines and examples regarding the distinction of (vertebrate or human) “primary cells” and “immortal cell lines”. Annotators will receive adequate training and will be obliged to use the Cellosaurus [22] for the identification of the cell lines. There also will be more emphasis on the hierarchical position of label “others” which actually is at the top of a hierarchical system, and captures abstracts that describe biomedical research that uses other than experimental approaches, e.g. observational studies.

We carried out annotation only on abstract-level, and even though the annotators highlighted some text passages in the TeamTat annotation tool, we did not require them to highlight all mentions of a particular model. As future work, we plan to consider these annotations for further experiments, as an extension of the preliminary sentence-based classification that we carried out.

We integrated the best performing BioBERT model in our SMAFIRA Web tool, which is currently under development. SMAFIRA is a search engine that aims at supporting researchers when searching for alternative methods to animal experiments. Our classifier based on PubMedBERT provides a real-time classification of the abstracts which are automatically retrieved from PubMed based on the user input query. This allows the user to filter the list of results based on the one or more of our labels.

## Conclusion

In this publication, we presented a new corpus of 1,600 Medline abstracts and manually annotated it using a set of eight labels (in vivo, organs, primary cells, immortal cell lines, human, invertebrates, in silico, and others). We obtained more than 7,000 annotations (multi-labeling was possible) and involved 13 annotators in the process (two annotations per document). After the first round of annotation, we achieved different degrees of agreement, and even a strong agreement for one of the labels. All disagreements were resolved in the additional annotation rounds. We expect that this corpus can support the development of applications in the field of alternative methods to animal experiments, as well as serve as a benchmark for biomedical text classification tasks.

We ran machine learning experiments to assess the feasibility of using our corpus to predict the labels. These experiments aimed at the identification of the best algorithm, hyperparameters, language model, and semantic features, such as MeSH terms and discourse elements. Further, we compared our proposed model to state-of-the-art tools for the prediction of named-entity recognition.

We provided an adequate analysis of our annotations and of the disagreements between the annotators. These insights will certainly be valuable when designing the next version of our corpus. Further, we investigated the correlation of our annotations with respect to the original queries, which were used to retrieve the abstracts, and the MeSH terms associated to the articles (cf. Section “Correlation with the initial database queries and the corresponding MeSH terms”). These results support our claim that queries based on MeSH terms are not adequate for precisely identifying the experimental method (or model) described in a publication.

### Supplementary Information

**Additional file 1.** Supplementary material. Queries for retrieval of the abstracts. Annotation guidelines. Analysis of the MeSH terms. Experiments with section labels. PMIDs removed in the second rounds. Statistics of the corpus. Results for Support Vector Machines. Selection of the hyperparameters. Annotations - error analysis.

## Data Availability

The dataset with the annotations was uploaded as supplementary material....

## Footnotes

1 https://eur-lex.europa.eu/legal-content/EN/TXT/?uri=CELEX:32010L0063

2 https://www.nal.usda.gov/animal-health-and-welfare/animal-welfare-act

3 https://pubmed.ncbi.nlm.nih.gov/

4 https://eutils.ncbi.nlm.nih.gov

5 https://ntp.niehs.nih.gov/go/altbib

6 https://www.nat-database.org/

7 https://www.open3r.org/

8 https://www.teamtat.org/

9 https://web.expasy.org/cellosaurus/

10 https://www.atcc.org/

11 based on “reliability”, i.e. high average agreement with the other annotators

12 https://github.com/huggingface/transformers

13 https://huggingface.co/dmis-lab/biobert-v1.1

14 https://huggingface.co/microsoft/BiomedNLP-PubMedBERT-base-uncased-abstract

15 https://huggingface.co/allenai/scibert_scivocab_uncased

16 https://huggingface.co/bionlp/bluebert_pubmed_mimic_uncased_L-12_H-768_A-12

17 https://github.com/anlausch/ArguminSci

18 The upper limit for any label thus is 2 x 1,600 (abstracts) = 3200, if a label was assigned to all abstracts in the corpus by both allotted annotators.

19 https://microsoft.github.io/BLURB/leaderboard.html

20 in particular “in vivo” models and models using biopsies or tissues from animals killed in advance (“ex vivo”)

21 with a frequency of at least 50
