# Supplementary material for: Automatic classification of experimental models in biomedical literature to support searching for alternative methods to animal experiments
Source: J Biomed Semantics. 2023 Sep 1;14:13. doi: 10.1186/s13326-023-00292-w (PMC10472567; doi:10.1186/s13326-023-00292-w)
Supplement: Supplementary file 1 — Additional file 1. Supplementary material. Queries for retrieval of the abstracts. Annotation guidelines. Analysis of the MeSH terms. Experiments with section labels. PMIDs removed in the second rounds. Statistics of the corpus. Results for Support Vector Machines. Selection of the hyperparameters. Annotations - error analysis. [file 13326_2023_292_MOESM1_ESM.pdf]

Supplementary material for the publication:

Automatic classification of experimental models in biomedical literature to support searching for alternative methods to animal experiments

Mariana Neves, Antonina Klippert, Fanny Knöspel, Juliane Rudeck, Ailine Stolz, Zsofia Ban, Markus Becker, Kai Diederich, Barbara Grune, Pia Kahnau, Nils Ohnesorge, Johannes Pucher, Gilbert Schönfelder, Bettina Bert and Daniel Butzke

Contents

1 Queries for retrieval of the abstracts 3

2 Annotation guidelines 16
2.1 In vivo experiments on vertebrates, cephalopods 17
2.2 Vertebrate organs and tissues 18
2.3 Vertebrate primary and stem cells 18
2.4 Vertebrate immortalized (transformed) and cancer cell lines . . . 18
2.5 Experiments with humans or human materials 18
2.6 Experiments with invertebrates or invertebrate materials 19
2.7 In silico 19
2.8 Other 19

3 Analysis of the MeSH terms 21

4 Experiments with section labels 22

5 PMIDs removed in the second rounds 23

6 Statistics of the corpus 24

7 Selection of the hyperparameters 25

8 Results for Support Vector Machines 29

9 Evaluation of NER tools 30

10 Annotations - error analysis **31**

1 Queries for retrieval of the abstracts

"In Vivo" ((”Animals, Newborn”[MeSH Terms] OR ”Disease Models, Animal”[MeSH Terms] OR ”Animals, Genetically Modified”[MeSH Terms] OR ”Animal Experimentation”[MeSH Terms] OR ”Vivisection”[MeSH Terms] OR ”Models, Animal”[MeSH Terms] OR ”Xenograft Model Antitumor Assays”[MeSH Terms] OR ”Neoplasm Transplantation”[MeSH Terms] OR ”Leukemia, Experimental”[MeSH Terms] OR ”Liver Neoplasms, Experimental”[MeSH Terms] OR ”Mammary Neoplasms, Experimental”[MeSH Terms] OR ”Melanoma, Experimental”[MeSH Terms] OR ”Sarcoma, Experimental”[MeSH Terms] OR ”Encephalomyelitis, Autoimmune, Experimental”[MeSH Terms] OR ”Myasthenia Gravis, Autoimmune, Experimental”[MeSH Terms] OR ”Neuritis, Autoimmune, Experimental”[MeSH Terms] OR ”Arthritis, Experimental”[MeSH Terms] OR ”Diabetes Mellitus, Experimental”[MeSH Terms] OR ”Liver Cirrhosis, Experimental”[MeSH Terms] OR ”Neoplasms, Experimental”[MeSH Terms] OR ”Nervous System Autoimmune Disease, Experimental”[MeSH Terms] OR ”Radiation Injuries, Experimental”[MeSH Terms] OR ”Mice”[MeSH Terms] OR ”Mice, Knockout”[MeSH Terms] OR ”Mice, Transgenic”[MeSH Terms] OR ”Mice, Inbred C57BL”[MeSH Terms] OR ”Mice, Nude”[MeSH Terms] OR ”Mice, SCID”[MeSH Terms] OR ”Mice, Congenic”[MeSH Terms] OR ”Mice, Inbred Strains”[MeSH Terms] OR ”Mice, Mutant Strains”[MeSH Terms] OR ”Mice, Obese”[MeSH Terms] OR ”Mice, 129 Strain”[MeSH Terms] OR ”Mice, Hairless”[MeSH Terms] OR ”Rats”[MeSH Terms] OR ”Rats, Sprague-Dawley”[MeSH Terms] OR ”Rats, Wistar”[MeSH Terms] OR ”Rats, Transgenic”[MeSH Terms] OR ”Rats, Mutant Strains”[MeSH Terms] OR ”Rats, Long-Evans”[MeSH Terms] OR ”Rats, Inbred Strains”[MeSH Terms]) AND ”Diseases Category”[MeSH Terms]) NOT (”Clinical Study”[Publication Type] OR ”Clinical Trial”[Publication Type] OR ”Clinical Trial, Phase I”[Publication Type] OR ”Clinical Trial, Phase II”[Publication Type] OR ”Clinical Trial, Phase III”[Publication Type] OR ”Clinical Trial, Phase IV”[Publication Type] OR ”Clinical Trial, Veterinary”[Publication Type] OR ”Clinical Trial Protocol”[Publication Type] OR ”Controlled Clinical Trial”[Publication Type] OR ”Clinical Conference”[Publication Type] OR ”Review”[Publication Type]) NOT (”Organ Culture Techniques”[MeSH Terms] OR ”Tissue Culture Techniques”[MeSH Terms] OR ”Tissue Extracts”[MeSH Terms] OR ”Actihaemyl”[MeSH Terms] OR ”Cell Extracts”[MeSH Terms] OR ”Liver Extracts”[MeSH Terms] OR ”Pancreatic Extracts”[MeSH Terms] OR ”Pancreatin”[MeSH Terms] OR ”Pancrelipase”[MeSH Terms] OR ”Placental Extracts”[MeSH Terms] OR ”Thymus Extracts”[MeSH Terms] OR ”Isolated Heart Preparation”[MeSH Terms]) NOT (”Adult Germline Stem Cells”[MeSH Terms] OR ”Adult Stem Cells”[MeSH Terms] OR ”Blastomeres”[MeSH Terms] OR ”Cellular Reprogramming Techniques”[MeSH Terms] OR ”Embryoid Bodies”[MeSH Terms] OR ”Embryonal Carcinoma Stem Cells”[MeSH Terms] OR ”Embryonal Carcinoma Stem Cells”[MeSH Terms] OR ”Embryonic Germ Cells”[MeSH Terms] OR ”Embryonic Stem Cells”[MeSH Terms] OR ”Fetal Stem Cells”[MeSH Terms] OR ”Granulocyte-Macrophage Progenitor Cells”[MeSH Terms] OR ”Hematopoietic Stem Cells”[MeSH Terms] OR ”Human Embryonic Stem Cells”[MeSH Terms] OR ”Induced Pluripotent

Stem Cells"[MeSH Terms] OR "Lymphoid Progenitor Cells"[MeSH Terms] OR "Megakaryocyte-Erythroid Progenitor Cells"[MeSH Terms] OR "Mesenchymal Stem Cells"[MeSH Terms] OR "Mouse Embryonic Stem Cells"[MeSH Terms] OR "Multipotent Stem Cells"[MeSH Terms] OR "Myeloid Progenitor Cells"[MeSH Terms] OR "Myoblasts, Cardiac"[MeSH Terms] OR "Myoblasts, Skeletal"[MeSH Terms] OR "Myoblasts, Smooth Muscle"[MeSH Terms] OR "Myoblasts"[MeSH Terms] OR "Neoplastic Stem Cells"[MeSH Terms] OR "Neural Stem Cells"[MeSH Terms] OR "Oligodendrocyte Precursor Cells"[MeSH Terms] OR "Oogonial Stem Cells"[MeSH Terms] OR "Peripheral Blood Stem Cells"[MeSH Terms] OR "Pluripotent Stem Cells"[MeSH Terms] OR "Precursor Cells, B-Lymphoid"[MeSH Terms] OR "Precursor Cells, T-Lymphoid"[MeSH Terms] OR "Primary Cell Culture"[MeSH Terms] OR "Satellite Cells, Skeletal Muscle"[MeSH Terms] OR "Side-Population Cells"[MeSH Terms] OR "Stem Cell Research"[MeSH Terms] OR "Stem Cells"[MeSH Terms] OR "Thymocytes"[MeSH Terms] OR "Totipotent Stem Cells"[MeSH Terms]) NOT ("3T3 Cells"[MeSH Terms] OR "A549 Cells"[MeSH Terms] OR "BALB 3T3 Cells"[MeSH Terms] OR "Caco-2 Cells"[MeSH Terms] OR "Cell Line, Transformed"[MeSH Terms] OR "Cell Line, Tumor"[MeSH Terms] OR "Cell Line"[All Fields] OR "CHO Cells"[MeSH Terms] OR "COS Cells"[MeSH Terms] OR "HCT116 Cells"[MeSH Terms] OR "HEK293 Cells"[MeSH Terms] OR "HeLa Cells"[MeSH Terms] OR "Hep G2 Cells"[MeSH Terms] OR "HL-60 Cells"[MeSH Terms] OR "HT29 Cells"[MeSH Terms] OR "Jurkat Cells"[MeSH Terms] OR "K562 Cells"[MeSH Terms] OR "L Cells (Cell Line)"[MeSH Terms] OR "LLC-PK1 Cells"[MeSH Terms] OR "Madin Darby Canine Kidney Cells"[MeSH Terms] OR "MCF-7 Cells"[MeSH Terms] OR "NIH 3T3 Cells"[MeSH Terms] OR "PC12 Cells"[MeSH Terms] OR "PC-3 Cells"[MeSH Terms] OR "RAW 264.7 Cells"[MeSH Terms] OR "Sf9 Cells"[MeSH Terms] OR "Swiss 3T3 Cells"[MeSH Terms] OR "THP-1 Cells"[MeSH Terms] OR "Tumor Cells, Cultured"[MeSH Terms] OR "Cells, Cultured"[MeSH Terms] OR "U937 Cells"[MeSH Terms] OR "Vero Cells"[MeSH Terms]) NOT "Computer Simulation"[MeSH Terms] NOT "Humans"[MeSH Terms:noexp] AND (hasabstract[text] AND "Animals"[MeSH Terms:noexp] AND English[lang])

"Organs/Tissues" (("Organ Culture Techniques"[MeSH Terms] OR "Tissue Culture Techniques"[MeSH Terms] OR "Tissue Extracts"[MeSH Terms] OR "Actihaemyl"[MeSH Terms] OR "Cell Extracts"[MeSH Terms] OR "Liver Extracts"[MeSH Terms] OR "Pancreatic Extracts"[MeSH Terms] OR "Pancreatin"[MeSH Terms] OR "Pancrelipase"[MeSH Terms] OR "Placental Extracts"[MeSH Terms] OR "Thymus Extracts"[MeSH Terms] OR "Isolated Heart Preparation"[MeSH Terms]) AND "Diseases Category"[MeSH Terms]) NOT ("Clinical Study"[Publication Type] OR "Clinical Trial"[Publication Type] OR "Clinical Trial, Phase I"[Publication Type] OR "Clinical Trial, Phase II"[Publication Type] OR "Clinical Trial, Phase III"[Publication Type] OR "Clinical Trial, Phase IV"[Publication Type] OR "Clinical Trial, Veterinary"[Publication Type] OR "Clinical Trial Protocol"[Publication Type] OR "Controlled Clinical Trial"[Publication Type] OR "Clinical Conference"[Publication Type] OR "Review"[Publication

Type]) NOT ("Animals, Newborn"[MeSH Terms] OR "Disease Models, Animal"[MeSH Terms] OR "Animals, Genetically Modified"[MeSH Terms] OR "Animal Experimentation"[MeSH Terms] OR "Vivisection"[MeSH Terms] OR "Models, Animal"[MeSH Terms] OR "Xenograft Model Antitumor Assays"[MeSH Terms] OR "Neoplasm Transplantation"[MeSH Terms] OR "Leukemia, Experimental"[MeSH Terms] OR "Liver Neoplasms, Experimental"[MeSH Terms] OR "Mammary Neoplasms, Experimental"[MeSH Terms] OR "Melanoma, Experimental"[MeSH Terms] OR "Sarcoma, Experimental"[MeSH Terms] OR "Encephalomyelitis, Autoimmune, Experimental"[MeSH Terms] OR "Myasthenia Gravis, Autoimmune, Experimental"[MeSH Terms] OR "Neuritis, Autoimmune, Experimental"[MeSH Terms] OR "Arthritis, Experimental"[MeSH Terms] OR "Diabetes Mellitus, Experimental"[MeSH Terms] OR "Liver Cirrhosis, Experimental"[MeSH Terms] OR "Neoplasms, Experimental"[MeSH Terms] OR "Nervous System Autoimmune Disease, Experimental"[MeSH Terms] OR "Radiation Injuries, Experimental"[MeSH Terms] OR "Mice"[MeSH Terms] OR "Mice, Knockout"[MeSH Terms] OR "Mice, Transgenic"[MeSH Terms] OR "Mice, Inbred C57BL"[MeSH Terms] OR "Mice, Nude"[MeSH Terms] OR "Mice, SCID"[MeSH Terms] OR "Mice, Congenic"[MeSH Terms] OR "Mice, Inbred Strains"[MeSH Terms] OR "Mice, Mutant Strains"[MeSH Terms] OR "Mice, Obese"[MeSH Terms] OR "Mice, 129 Strain"[MeSH Terms] OR "Mice, Hairless"[MeSH Terms] OR "Rats"[MeSH Terms] OR "Rats, Sprague-Dawley"[MeSH Terms] OR "Rats, Wistar"[MeSH Terms] OR "Rats, Transgenic"[MeSH Terms] OR "Rats, Mutant Strains"[MeSH Terms] OR "Rats, Long-Evans"[MeSH Terms] OR "Rats, Inbred Strains"[MeSH Terms]) NOT ("Adult Germline Stem Cells"[MeSH Terms] OR "Adult Stem Cells"[MeSH Terms] OR "Blastomeres"[MeSH Terms] OR "Cellular Reprogramming Techniques"[MeSH Terms] OR "Embryoid Bodies"[MeSH Terms] OR "Embryonal Carcinoma Stem Cells"[MeSH Terms] OR "Embryonal Carcinoma Stem Cells"[MeSH Terms] OR "Embryonic Germ Cells"[MeSH Terms] OR "Embryonic Stem Cells"[MeSH Terms] OR "Fetal Stem Cells"[MeSH Terms] OR "Granulocyte-Macrophage Progenitor Cells"[MeSH Terms] OR "Hematopoietic Stem Cells"[MeSH Terms] OR "Human Embryonic Stem Cells"[MeSH Terms] OR "Induced Pluripotent Stem Cells"[MeSH Terms] OR "Lymphoid Progenitor Cells"[MeSH Terms] OR "Megakaryocyte-Erythroid Progenitor Cells"[MeSH Terms] OR "Mesenchymal Stem Cells"[MeSH Terms] OR "Mouse Embryonic Stem Cells"[MeSH Terms] OR "Multipotent Stem Cells"[MeSH Terms] OR "Myeloid Progenitor Cells"[MeSH Terms] OR "Myoblasts, Cardiac"[MeSH Terms] OR "Myoblasts, Skeletal"[MeSH Terms] OR "Myoblasts, Smooth Muscle"[MeSH Terms] OR "Myoblasts"[MeSH Terms] OR "Neoplastic Stem Cells"[MeSH Terms] OR "Neural Stem Cells"[MeSH Terms] OR "Oligodendrocyte Precursor Cells"[MeSH Terms] OR "Oogonial Stem Cells"[MeSH Terms] OR "Peripheral Blood Stem Cells"[MeSH Terms] OR "Pluripotent Stem Cells"[MeSH Terms] OR "Precursor Cells, B-Lymphoid"[MeSH Terms] OR "Precursor Cells, T-Lymphoid"[MeSH Terms] OR "Primary Cell Culture"[MeSH Terms] OR "Satellite Cells, Skeletal Muscle"[MeSH Terms] OR "Side-Population Cells"[MeSH Terms] OR "Stem Cell Research"[MeSH Terms] OR "Stem Cells"[MeSH Terms] OR "Thymocytes"[MeSH Terms] OR "Totipotent Stem Cells"[MeSH Terms]) NOT ("3T3

Cells"[MeSH Terms] OR "A549 Cells"[MeSH Terms] OR "BALB 3T3 Cells"[MeSH Terms] OR "Caco-2 Cells"[MeSH Terms] OR "Cell Line, Transformed"[MeSH Terms] OR "Cell Line, Tumor"[MeSH Terms] OR "Cell Line"[All Fields] OR "CHO Cells"[MeSH Terms] OR "COS Cells"[MeSH Terms] OR "HCT116 Cells"[MeSH Terms] OR "HEK293 Cells"[MeSH Terms] OR "HeLa Cells"[MeSH Terms] OR "Hep G2 Cells"[MeSH Terms] OR "HL-60 Cells"[MeSH Terms] OR "HT29 Cells"[MeSH Terms] OR "Jurkat Cells"[MeSH Terms] OR "K562 Cells"[MeSH Terms] OR "L Cells (Cell Line)"[MeSH Terms] OR "LLC-PK1 Cells"[MeSH Terms] OR "Madin Darby Canine Kidney Cells"[MeSH Terms] OR "MCF-7 Cells"[MeSH Terms] OR "NIH 3T3 Cells"[MeSH Terms] OR "PC12 Cells"[MeSH Terms] OR "PC-3 Cells"[MeSH Terms] OR "RAW 264.7 Cells"[MeSH Terms] OR "Sf9 Cells"[MeSH Terms] OR "Swiss 3T3 Cells"[MeSH Terms] OR "THP-1 Cells"[MeSH Terms] OR "Tumor Cells, Cultured"[MeSH Terms] OR "Cells, Cultured"[MeSH Terms] OR "U937 Cells"[MeSH Terms] OR "Vero Cells"[MeSH Terms]) NOT "Computer Simulation"[MeSH Terms] NOT "Humans"[MeSH Terms:noexp] AND (hasabstract[text] AND "animals"[MeSH Terms:noexp] AND English[lang])

"Primary and Stem Cells" (("Adult Germline Stem Cells"[MeSH Terms] OR "Adult Stem Cells"[MeSH Terms] OR "Blastomeres"[MeSH Terms] OR "Cellular Reprogramming Techniques"[MeSH Terms] OR "Embryoid Bodies"[MeSH Terms] OR "Embryonal Carcinoma Stem Cells"[MeSH Terms] OR "Embryonal Carcinoma Stem Cells"[MeSH Terms] OR "Embryonic Germ Cells"[MeSH Terms] OR "Embryonic Stem Cells"[MeSH Terms] OR "Fetal Stem Cells"[MeSH Terms] OR "Granulocyte-Macrophage Progenitor Cells"[MeSH Terms] OR "Hematopoietic Stem Cells"[MeSH Terms] OR "Human Embryonic Stem Cells"[MeSH Terms] OR "Induced Pluripotent Stem Cells"[MeSH Terms] OR "Lymphoid Progenitor Cells"[MeSH Terms] OR "Megakaryocyte-Erythroid Progenitor Cells"[MeSH Terms] OR "Mesenchymal Stem Cells"[MeSH Terms] OR "Mouse Embryonic Stem Cells"[MeSH Terms] OR "Multipotent Stem Cells"[MeSH Terms] OR "Myeloid Progenitor Cells"[MeSH Terms] OR "Myoblasts, Cardiac"[MeSH Terms] OR "Myoblasts, Skeletal"[MeSH Terms] OR "Myoblasts, Smooth Muscle"[MeSH Terms] OR "Myoblasts"[MeSH Terms] OR "Neoplastic Stem Cells"[MeSH Terms] OR "Neural Stem Cells"[MeSH Terms] OR "Oligodendrocyte Precursor Cells"[MeSH Terms] OR "Oogonial Stem Cells"[MeSH Terms] OR "Peripheral Blood Stem Cells"[MeSH Terms] OR "Pluripotent Stem Cells"[MeSH Terms] OR "Precursor Cells, B-Lymphoid"[MeSH Terms] OR "Precursor Cells, T-Lymphoid"[MeSH Terms] OR "Primary Cell Culture"[MeSH Terms] OR "Satellite Cells, Skeletal Muscle"[MeSH Terms] OR "Side-Population Cells"[MeSH Terms] OR "Stem Cell Research"[MeSH Terms] OR "Stem Cells"[MeSH Terms] OR "Thymocytes"[MeSH Terms] OR "Totipotent Stem Cells"[MeSH Terms]) AND "Diseases Category"[MeSH Terms]) NOT ("Clinical Study"[Publication Type] OR "Clinical Trial"[Publication Type] OR "Clinical Trial, Phase I"[Publication Type] OR "Clinical Trial, Phase II"[Publication Type] OR "Clinical Trial, Phase III"[Publication Type] OR "Clinical Trial, Phase IV"[Publication Type]

OR "Clinical Trial, Veterinary"[Publication Type] OR "Clinical Trial Protocol"[Publication Type] OR "Controlled Clinical Trial"[Publication Type] OR "Clinical Conference"[Publication Type] OR "Review"[Publication Type]) NOT ("Animals, Newborn"[MeSH Terms] OR "Disease Models, Animal"[MeSH Terms] OR "Animals, Genetically Modified"[MeSH Terms] OR "Animal Experimentation"[MeSH Terms] OR "Vivisection"[MeSH Terms] OR "Models, Animal"[MeSH Terms] OR "Xenograft Model Antitumor Assays"[MeSH Terms] OR "Neoplasm Transplantation"[MeSH Terms] OR "Leukemia, Experimental"[MeSH Terms] OR "Liver Neoplasms, Experimental"[MeSH Terms] OR "Mammary Neoplasms, Experimental"[MeSH Terms] OR "Melanoma, Experimental"[MeSH Terms] OR "Sarcoma, Experimental"[MeSH Terms] OR "Encephalomyelitis, Autoimmune, Experimental"[MeSH Terms] OR "Myasthenia Gravis, Autoimmune, Experimental"[MeSH Terms] OR "Neuritis, Autoimmune, Experimental"[MeSH Terms] OR "Arthritis, Experimental"[MeSH Terms] OR "Diabetes Mellitus, Experimental"[MeSH Terms] OR "Liver Cirrhosis, Experimental"[MeSH Terms] OR "Neoplasms, Experimental"[MeSH Terms] OR "Nervous System Autoimmune Disease, Experimental"[MeSH Terms] OR "Radiation Injuries, Experimental"[MeSH Terms] OR "Mice"[MeSH Terms] OR "Mice, Knockout"[MeSH Terms] OR "Mice, Transgenic"[MeSH Terms] OR "Mice, Inbred C57BL"[MeSH Terms] OR "Mice, Nude"[MeSH Terms] OR "Mice, SCID"[MeSH Terms] OR "Mice, Congenic"[MeSH Terms] OR "Mice, Inbred Strains"[MeSH Terms] OR "Mice, Mutant Strains"[MeSH Terms] OR "Mice, Obese"[MeSH Terms] OR "Mice, 129 Strain"[MeSH Terms] OR "Mice, Hairless"[MeSH Terms] OR "Rats"[MeSH Terms] OR "Rats, Sprague-Dawley"[MeSH Terms] OR "Rats, Wistar"[MeSH Terms] OR "Rats, Transgenic"[MeSH Terms] OR "Rats, Mutant Strains"[MeSH Terms] OR "Rats, Long-Evans"[MeSH Terms] OR "Rats, Inbred Strains"[MeSH Terms]) NOT ("Organ Culture Techniques"[MeSH Terms] OR "Tissue Culture Techniques"[MeSH Terms] OR "Tissue Extracts"[MeSH Terms] OR "Actihaemyl"[MeSH Terms] OR "Cell Extracts"[MeSH Terms] OR "Liver Extracts"[MeSH Terms] OR "Pancreatic Extracts"[MeSH Terms] OR "Pancreatin"[MeSH Terms] OR "Pancrelipase"[MeSH Terms] OR "Placental Extracts"[MeSH Terms] OR "Thymus Extracts"[MeSH Terms] OR "Isolated Heart Preparation"[MeSH Terms]) NOT ("3T3 Cells"[MeSH Terms] OR "A549 Cells"[MeSH Terms] OR "BALB 3T3 Cells"[MeSH Terms] OR "Caco-2 Cells"[MeSH Terms] OR "Cell Line, Transformed"[MeSH Terms] OR "Cell Line, Tumor"[MeSH Terms] OR "Cell Line"[All Fields] OR "CHO Cells"[MeSH Terms] OR "COS Cells"[MeSH Terms] OR "HCT116 Cells"[MeSH Terms] OR "HEK293 Cells"[MeSH Terms] OR "HeLa Cells"[MeSH Terms] OR "Hep G2 Cells"[MeSH Terms] OR "HL-60 Cells"[MeSH Terms] OR "HT29 Cells"[MeSH Terms] OR "Jurkat Cells"[MeSH Terms] OR "K562 Cells"[MeSH Terms] OR "L Cells (Cell Line)"[MeSH Terms] OR "LLC-PK1 Cells"[MeSH Terms] OR "Madin Darby Canine Kidney Cells"[MeSH Terms] OR "MCF-7 Cells"[MeSH Terms] OR "NIH 3T3 Cells"[MeSH Terms] OR "PC12 Cells"[MeSH Terms] OR "PC-3 Cells"[MeSH Terms] OR "RAW 264.7 Cells"[MeSH Terms] OR "Sf9 Cells"[MeSH Terms] OR "Swiss 3T3 Cells"[MeSH Terms] OR "THP-1 Cells"[MeSH Terms] OR "Tumor Cells, Cultured"[MeSH Terms] OR "Cells, Cultured"[MeSH Terms] OR "U937 Cells"[MeSH Terms] OR "Vero Cells"[MeSH

Terms]) NOT "Computer Simulation"[MeSH Terms] NOT "Humans"[MeSH Terms:noexp] AND (hasabstract[text] AND "animals"[MeSH Terms:noexp] AND English[lang])

"Immortal/Tumor Cell Lines" (("3T3 Cells"[MeSH Terms] OR "A549 Cells"[MeSH Terms] OR "BALB 3T3 Cells"[MeSH Terms] OR "Caco-2 Cells"[MeSH Terms] OR "Cell Line, Transformed"[MeSH Terms] OR "Cell Line, Tumor"[MeSH Terms] OR "Cell Line"[All Fields] OR "CHO Cells"[MeSH Terms] OR "COS Cells"[MeSH Terms] OR "HCT116 Cells"[MeSH Terms] OR "HEK293 Cells"[MeSH Terms] OR "HeLa Cells"[MeSH Terms] OR "Hep G2 Cells"[MeSH Terms] OR "HL-60 Cells"[MeSH Terms] OR "HT29 Cells"[MeSH Terms] OR "Jurkat Cells"[MeSH Terms] OR "K562 Cells"[MeSH Terms] OR "L Cells (Cell Line)"[MeSH Terms] OR "LLC-PK1 Cells"[MeSH Terms] OR "Madin Darby Canine Kidney Cells"[MeSH Terms] OR "MCF-7 Cells"[MeSH Terms] OR "NIH 3T3 Cells"[MeSH Terms] OR "PC12 Cells"[MeSH Terms] OR "PC-3 Cells"[MeSH Terms] OR "RAW 264.7 Cells"[MeSH Terms] OR "Sf9 Cells"[MeSH Terms] OR "Swiss 3T3 Cells"[MeSH Terms] OR "THP-1 Cells"[MeSH Terms] OR "Tumor Cells, Cultured"[MeSH Terms] OR "Cells, Cultured"[MeSH Terms] OR "U937 Cells"[MeSH Terms] OR "Vero Cells"[MeSH Terms]) AND "Diseases Category"[MeSH Terms]) NOT ("Clinical Study"[Publication Type] OR "Clinical Trial"[Publication Type] OR "Clinical Trial, Phase I"[Publication Type] OR "Clinical Trial, Phase II"[Publication Type] OR "Clinical Trial, Phase III"[Publication Type] OR "Clinical Trial, Phase IV"[Publication Type] OR "Clinical Trial, Veterinary"[Publication Type] OR "Clinical Trial Protocol"[Publication Type] OR "Controlled Clinical Trial"[Publication Type] OR "Clinical Conference"[Publication Type] OR "Review"[Publication Type]) NOT ("Animals, Newborn"[MeSH Terms] OR "Disease Models, Animal"[MeSH Terms] OR "Animals, Genetically Modified"[MeSH Terms] OR "Animal Experimentation"[MeSH Terms] OR "Vivisection"[MeSH Terms] OR "Models, Animal"[MeSH Terms] OR "Xenograft Model Antitumor Assays"[MeSH Terms] OR "Neoplasm Transplantation"[MeSH Terms] OR "Leukemia, Experimental"[MeSH Terms] OR "Liver Neoplasms, Experimental"[MeSH Terms] OR "Mammary Neoplasms, Experimental"[MeSH Terms] OR "Melanoma, Experimental"[MeSH Terms] OR "Sarcoma, Experimental"[MeSH Terms] OR "Encephalomyelitis, Autoimmune, Experimental"[MeSH Terms] OR "Myasthenia Gravis, Autoimmune, Experimental"[MeSH Terms] OR "Neuritis, Autoimmune, Experimental"[MeSH Terms] OR "Arthritis, Experimental"[MeSH Terms] OR "Diabetes Mellitus, Experimental"[MeSH Terms] OR "Liver Cirrhosis, Experimental"[MeSH Terms] OR "Neoplasms, Experimental"[MeSH Terms] OR "Nervous System Autoimmune Disease, Experimental"[MeSH Terms] OR "Radiation Injuries, Experimental"[MeSH Terms] OR "Mice"[MeSH Terms] OR "Mice, Knockout"[MeSH Terms] OR "Mice, Transgenic"[MeSH Terms] OR "Mice, Inbred C57BL"[MeSH Terms] OR "Mice, Nude"[MeSH Terms] OR "Mice, SCID"[MeSH Terms] OR "Mice, Congenic"[MeSH Terms] OR "Mice, Inbred Strains"[MeSH Terms] OR "Mice, Mutant Strains"[MeSH Terms] OR "Mice, Obese"[MeSH Terms] OR "Mice, 129 Strain"[MeSH Terms] OR "Mice, Hairless"[MeSH Terms]

OR "Rats"[MeSH Terms] OR "Rats, Sprague-Dawley"[MeSH Terms] OR "Rats, Wistar"[MeSH Terms] OR "Rats, Transgenic"[MeSH Terms] OR "Rats, Mutant Strains"[MeSH Terms] OR "Rats, Long-Evans"[MeSH Terms] OR "Rats, Inbred Strains"[MeSH Terms]) NOT ("Organ Culture Techniques"[MeSH Terms] OR "Tissue Culture Techniques"[MeSH Terms] OR "Tissue Extracts"[MeSH Terms] OR "Actihaemyl"[MeSH Terms] OR "Cell Extracts"[MeSH Terms] OR "Liver Extracts"[MeSH Terms] OR "Pancreatic Extracts"[MeSH Terms] OR "Pancreatin"[MeSH Terms] OR "Pancrelipase"[MeSH Terms] OR "Placental Extracts"[MeSH Terms] OR "Thymus Extracts"[MeSH Terms] OR "Isolated Heart Preparation"[MeSH Terms]) NOT ("Adult Germline Stem Cells"[MeSH Terms] OR "Adult Stem Cells"[MeSH Terms] OR "Blastomeres"[MeSH Terms] OR "Cellular Reprogramming Techniques"[MeSH Terms] OR "Embryoid Bodies"[MeSH Terms] OR "Embryonal Carcinoma Stem Cells"[MeSH Terms] OR "Embryonal Carcinoma Stem Cells"[MeSH Terms] OR "Embryonic Germ Cells"[MeSH Terms] OR "Embryonic Stem Cells"[MeSH Terms] OR "Fetal Stem Cells"[MeSH Terms] OR "Granulocyte-Macrophage Progenitor Cells"[MeSH Terms] OR "Hematopoietic Stem Cells"[MeSH Terms] OR "Human Embryonic Stem Cells"[MeSH Terms] OR "Induced Pluripotent Stem Cells"[MeSH Terms] OR "Lymphoid Progenitor Cells"[MeSH Terms] OR "Megakaryocyte-Erythroid Progenitor Cells"[MeSH Terms] OR "Mesenchymal Stem Cells"[MeSH Terms] OR "Mouse Embryonic Stem Cells"[MeSH Terms] OR "Multipotent Stem Cells"[MeSH Terms] OR "Myeloid Progenitor Cells"[MeSH Terms] OR "Myoblasts, Cardiac"[MeSH Terms] OR "Myoblasts, Skeletal"[MeSH Terms] OR "Myoblasts, Smooth Muscle"[MeSH Terms] OR "Myoblasts"[MeSH Terms] OR "Neoplastic Stem Cells"[MeSH Terms] OR "Neural Stem Cells"[MeSH Terms] OR "Oligodendrocyte Precursor Cells"[MeSH Terms] OR "Oogonial Stem Cells"[MeSH Terms] OR "Peripheral Blood Stem Cells"[MeSH Terms] OR "Pluripotent Stem Cells"[MeSH Terms] OR "Precursor Cells, B-Lymphoid"[MeSH Terms] OR "Precursor Cells, T-Lymphoid"[MeSH Terms] OR "Primary Cell Culture"[MeSH Terms] OR "Satellite Cells, Skeletal Muscle"[MeSH Terms] OR "Side-Population Cells"[MeSH Terms] OR "Stem Cell Research"[MeSH Terms] OR "Stem Cells"[MeSH Terms] OR "Thymocytes"[MeSH Terms] OR "Totipotent Stem Cells"[MeSH Terms]) NOT "Computer Simulation"[MeSH Terms] NOT "Humans"[MeSH Terms:noexp] AND (hasabstract[text] AND "animals"[MeSH Terms:noexp] AND English[lang])

"In Silico" ("Computer Simulation"[MeSH Terms] AND "Diseases Category"[MeSH Terms]) NOT ("Clinical Study"[Publication Type] OR "Clinical Trial"[Publication Type] OR "Clinical Trial, Phase I"[Publication Type] OR "Clinical Trial, Phase II"[Publication Type] OR "Clinical Trial, Phase III"[Publication Type] OR "Clinical Trial, Phase IV"[Publication Type] OR "Clinical Trial, Veterinary"[Publication Type] OR "Clinical Trial Protocol"[Publication Type] OR "Controlled Clinical Trial"[Publication Type] OR "Clinical Conference"[Publication Type] OR "Review"[Publication Type]) NOT ("Animals, Newborn"[MeSH Terms] OR "Disease Models, Animal"[MeSH Terms] OR "Animals, Genetically Modified"[MeSH Terms] OR "Animal Experimentation"[MeSH Terms] OR "Vivisection"[MeSH

Terms] OR "Models, Animal"[MeSH Terms] OR "Xenograft Model Antitumor Assays"[MeSH Terms] OR "Neoplasm Transplantation"[MeSH Terms] OR "Leukemia, Experimental"[MeSH Terms] OR "Liver Neoplasms, Experimental"[MeSH Terms] OR "Mammary Neoplasms, Experimental"[MeSH Terms] OR "Melanoma, Experimental"[MeSH Terms] OR "Sarcoma, Experimental"[MeSH Terms] OR "Encephalomyelitis, Autoimmune, Experimental"[MeSH Terms] OR "Myasthenia Gravis, Autoimmune, Experimental"[MeSH Terms] OR "Neuritis, Autoimmune, Experimental"[MeSH Terms] OR "Arthritis, Experimental"[MeSH Terms] OR "Diabetes Mellitus, Experimental"[MeSH Terms] OR "Liver Cirrhosis, Experimental"[MeSH Terms] OR "Neoplasms, Experimental"[MeSH Terms] OR "Nervous System Autoimmune Disease, Experimental"[MeSH Terms] OR "Radiation Injuries, Experimental"[MeSH Terms] OR "Mice"[MeSH Terms] OR "Mice, Knockout"[MeSH Terms] OR "Mice, Transgenic"[MeSH Terms] OR "Mice, Inbred C57BL"[MeSH Terms] OR "Mice, Nude"[MeSH Terms] OR "Mice, SCID"[MeSH Terms] OR "Mice, Congenic"[MeSH Terms] OR "Mice, Inbred Strains"[MeSH Terms] OR "Mice, Mutant Strains"[MeSH Terms] OR "Mice, Obese"[MeSH Terms] OR "Mice, 129 Strain"[MeSH Terms] OR "Mice, Hairless"[MeSH Terms] OR "Rats"[MeSH Terms] OR "Rats, Sprague-Dawley"[MeSH Terms] OR "Rats, Wistar"[MeSH Terms] OR "Rats, Transgenic"[MeSH Terms] OR "Rats, Mutant Strains"[MeSH Terms] OR "Rats, Long-Evans"[MeSH Terms] OR "Rats, Inbred Strains"[MeSH Terms]) NOT ("Organ Culture Techniques"[MeSH Terms] OR "Tissue Culture Techniques"[MeSH Terms] OR "Tissue Extracts"[MeSH Terms] OR "Actihaemyl"[MeSH Terms] OR "Cell Extracts"[MeSH Terms] OR "Liver Extracts"[MeSH Terms] OR "Pancreatic Extracts"[MeSH Terms] OR "Pancreatin"[MeSH Terms] OR "Pancrelipase"[MeSH Terms] OR "Placental Extracts"[MeSH Terms] OR "Thymus Extracts"[MeSH Terms] OR "Isolated Heart Preparation"[MeSH Terms]) NOT ("Adult Germline Stem Cells"[MeSH Terms] OR "Adult Stem Cells"[MeSH Terms] OR "Blastomeres"[MeSH Terms] OR "Cellular Reprogramming Techniques"[MeSH Terms] OR "Embryoid Bodies"[MeSH Terms] OR "Embryonal Carcinoma Stem Cells"[MeSH Terms] OR "Embryonal Carcinoma Stem Cells"[MeSH Terms] OR "Embryonic Germ Cells"[MeSH Terms] OR "Embryonic Stem Cells"[MeSH Terms] OR "Fetal Stem Cells"[MeSH Terms] OR "Granulocyte-Macrophage Progenitor Cells"[MeSH Terms] OR "Hematopoietic Stem Cells"[MeSH Terms] OR "Human Embryonic Stem Cells"[MeSH Terms] OR "Induced Pluripotent Stem Cells"[MeSH Terms] OR "Lymphoid Progenitor Cells"[MeSH Terms] OR "Megakaryocyte-Erythroid Progenitor Cells"[MeSH Terms] OR "Mesenchymal Stem Cells"[MeSH Terms] OR "Mouse Embryonic Stem Cells"[MeSH Terms] OR "Multipotent Stem Cells"[MeSH Terms] OR "Myeloid Progenitor Cells"[MeSH Terms] OR "Myoblasts, Cardiac"[MeSH Terms] OR "Myoblasts, Skeletal"[MeSH Terms] OR "Myoblasts, Smooth Muscle"[MeSH Terms] OR "Myoblasts"[MeSH Terms] OR "Neoplastic Stem Cells"[MeSH Terms] OR "Neural Stem Cells"[MeSH Terms] OR "Oligodendrocyte Precursor Cells"[MeSH Terms] OR "Oogonial Stem Cells"[MeSH Terms] OR "Peripheral Blood Stem Cells"[MeSH Terms] OR "Pluripotent Stem Cells"[MeSH Terms] OR "Precursor Cells, B-Lymphoid"[MeSH Terms] OR "Precursor Cells, T-Lymphoid"[MeSH Terms] OR "Primary Cell Culture"[MeSH Terms] OR "Satellite Cells, Skeletal

Muscle"[MeSH Terms] OR "Side-Population Cells"[MeSH Terms] OR "Stem Cell Research"[MeSH Terms] OR "Stem Cells"[MeSH Terms] OR "Thymocytes"[MeSH Terms] OR "Totipotent Stem Cells"[MeSH Terms]) NOT ("3T3 Cells"[MeSH Terms] OR "A549 Cells"[MeSH Terms] OR "BALB 3T3 Cells"[MeSH Terms] OR "Caco-2 Cells"[MeSH Terms] OR "Cell Line, Transformed"[MeSH Terms] OR "Cell Line, Tumor"[MeSH Terms] OR "Cell Line"[All Fields] OR "CHO Cells"[MeSH Terms] OR "COS Cells"[MeSH Terms] OR "HCT116 Cells"[MeSH Terms] OR "HEK293 Cells"[MeSH Terms] OR "HeLa Cells"[MeSH Terms] OR "Hep G2 Cells"[MeSH Terms] OR "HL-60 Cells"[MeSH Terms] OR "HT29 Cells"[MeSH Terms] OR "Jurkat Cells"[MeSH Terms] OR "K562 Cells"[MeSH Terms] OR "L Cells (Cell Line)"[MeSH Terms] OR "LLC-PK1 Cells"[MeSH Terms] OR "Madin Darby Canine Kidney Cells"[MeSH Terms] OR "MCF-7 Cells"[MeSH Terms] OR "NIH 3T3 Cells"[MeSH Terms] OR "PC12 Cells"[MeSH Terms] OR "PC-3 Cells"[MeSH Terms] OR "RAW 264.7 Cells"[MeSH Terms] OR "Sf9 Cells"[MeSH Terms] OR "Swiss 3T3 Cells"[MeSH Terms] OR "THP-1 Cells"[MeSH Terms] OR "Tumor Cells, Cultured"[MeSH Terms] OR "Cells, Cultured"[MeSH Terms] OR "U937 Cells"[MeSH Terms] OR "Vero Cells"[MeSH Terms]) NOT "Humans"[MeSH Terms:noexp] AND (hasabstract[text] AND "animals"[MeSH Terms:noexp] AND English[lang])

"Other" ("Humans"[MeSH Terms:noexp] AND "Diseases Category"[MeSH Terms]) NOT ("Clinical Study"[Publication Type] OR "Clinical Trial"[Publication Type] OR "Clinical Trial, Phase I"[Publication Type] OR "Clinical Trial, Phase II"[Publication Type] OR "Clinical Trial, Phase III"[Publication Type] OR "Clinical Trial, Phase IV"[Publication Type] OR "Clinical Trial, Veterinary"[Publication Type] OR "Clinical Trial Protocol"[Publication Type] OR "Controlled Clinical Trial"[Publication Type] OR "Clinical Conference"[Publication Type] OR "Review"[Publication Type]) NOT ("Animals, Newborn"[MeSH Terms] OR "Disease Models, Animal"[MeSH Terms] OR "Animals, Genetically Modified"[MeSH Terms] OR "Animal Experimentation"[MeSH Terms] OR "Vivisection"[MeSH Terms] OR "Models, Animal"[MeSH Terms] OR "Xenograft Model Antitumor Assays"[MeSH Terms] OR "Neoplasm Transplantation"[MeSH Terms] OR "Leukemia, Experimental"[MeSH Terms] OR "Liver Neoplasms, Experimental"[MeSH Terms] OR "Mammary Neoplasms, Experimental"[MeSH Terms] OR "Melanoma, Experimental"[MeSH Terms] OR "Sarcoma, Experimental"[MeSH Terms] OR "Encephalomyelitis, Autoimmune, Experimental"[MeSH Terms] OR "Myasthenia Gravis, Autoimmune, Experimental"[MeSH Terms] OR "Neuritis, Autoimmune, Experimental"[MeSH Terms] OR "Arthritis, Experimental"[MeSH Terms] OR "Diabetes Mellitus, Experimental"[MeSH Terms] OR "Liver Cirrhosis, Experimental"[MeSH Terms] OR "Neoplasms, Experimental"[MeSH Terms] OR "Nervous System Autoimmune Disease, Experimental"[MeSH Terms] OR "Radiation Injuries, Experimental"[MeSH Terms] OR "Mice"[MeSH Terms] OR "Mice, Knockout"[MeSH Terms] OR "Mice, Transgenic"[MeSH Terms] OR "Mice, Inbred C57BL"[MeSH Terms] OR "Mice, Nude"[MeSH Terms] OR "Mice, SCID"[MeSH Terms] OR "Mice, Congenic"[MeSH Terms] OR "Mice, Inbred Strains"[MeSH

Terms] OR "Mice, Mutant Strains"[MeSH Terms] OR "Mice, Obese"[MeSH Terms] OR "Mice, 129 Strain"[MeSH Terms] OR "Mice, Hairless"[MeSH Terms] OR "Rats"[MeSH Terms] OR "Rats, Sprague-Dawley"[MeSH Terms] OR "Rats, Wistar"[MeSH Terms] OR "Rats, Transgenic"[MeSH Terms] OR "Rats, Mutant Strains"[MeSH Terms] OR "Rats, Long-Evans"[MeSH Terms] OR "Rats, Inbred Strains"[MeSH Terms]) NOT ("Organ Culture Techniques"[MeSH Terms] OR "Tissue Culture Techniques"[MeSH Terms] OR "Tissue Extracts"[MeSH Terms] OR "Actihaemyl"[MeSH Terms] OR "Cell Extracts"[MeSH Terms] OR "Liver Extracts"[MeSH Terms] OR "Pancreatic Extracts"[MeSH Terms] OR "Pancreatin"[MeSH Terms] OR "Pancrelipase"[MeSH Terms] OR "Placental Extracts"[MeSH Terms] OR "Thymus Extracts"[MeSH Terms] OR "Isolated Heart Preparation"[MeSH Terms]) NOT ("Adult Germline Stem Cells"[MeSH Terms] OR "Adult Stem Cells"[MeSH Terms] OR "Blastomeres"[MeSH Terms] OR "Cellular Reprogramming Techniques"[MeSH Terms] OR "Embryoid Bodies"[MeSH Terms] OR "Embryonal Carcinoma Stem Cells"[MeSH Terms] OR "Embryonal Carcinoma Stem Cells"[MeSH Terms] OR "Embryonic Germ Cells"[MeSH Terms] OR "Embryonic Stem Cells"[MeSH Terms] OR "Fetal Stem Cells"[MeSH Terms] OR "Granulocyte-Macrophage Progenitor Cells"[MeSH Terms] OR "Hematopoietic Stem Cells"[MeSH Terms] OR "Human Embryonic Stem Cells"[MeSH Terms] OR "Induced Pluripotent Stem Cells"[MeSH Terms] OR "Lymphoid Progenitor Cells"[MeSH Terms] OR "Megakaryocyte-Erythroid Progenitor Cells"[MeSH Terms] OR "Mesenchymal Stem Cells"[MeSH Terms] OR "Mouse Embryonic Stem Cells"[MeSH Terms] OR "Multipotent Stem Cells"[MeSH Terms] OR "Myeloid Progenitor Cells"[MeSH Terms] OR "Myoblasts, Cardiac"[MeSH Terms] OR "Myoblasts, Skeletal"[MeSH Terms] OR "Myoblasts, Smooth Muscle"[MeSH Terms] OR "Myoblasts"[MeSH Terms] OR "Neoplastic Stem Cells"[MeSH Terms] OR "Neural Stem Cells"[MeSH Terms] OR "Oligodendrocyte Precursor Cells"[MeSH Terms] OR "Oogonial Stem Cells"[MeSH Terms] OR "Peripheral Blood Stem Cells"[MeSH Terms] OR "Pluripotent Stem Cells"[MeSH Terms] OR "Precursor Cells, B-Lymphoid"[MeSH Terms] OR "Precursor Cells, T-Lymphoid"[MeSH Terms] OR "Primary Cell Culture"[MeSH Terms] OR "Satellite Cells, Skeletal Muscle"[MeSH Terms] OR "Side-Population Cells"[MeSH Terms] OR "Stem Cell Research"[MeSH Terms] OR "Stem Cells"[MeSH Terms] OR "Thymocytes"[MeSH Terms] OR "Totipotent Stem Cells"[MeSH Terms]) NOT ("3T3 Cells"[MeSH Terms] OR "A549 Cells"[MeSH Terms] OR "BALB 3T3 Cells"[MeSH Terms] OR "Caco-2 Cells"[MeSH Terms] OR "Cell Line, Transformed"[MeSH Terms] OR "Cell Line, Tumor"[MeSH Terms] OR "Cell Line"[All Fields] OR "CHO Cells"[MeSH Terms] OR "COS Cells"[MeSH Terms] OR "HCT116 Cells"[MeSH Terms] OR "HEK293 Cells"[MeSH Terms] OR "HeLa Cells"[MeSH Terms] OR "Hep G2 Cells"[MeSH Terms] OR "HL-60 Cells"[MeSH Terms] OR "HT29 Cells"[MeSH Terms] OR "Jurkat Cells"[MeSH Terms] OR "K562 Cells"[MeSH Terms] OR "L Cells (Cell Line)"[MeSH Terms] OR "LLC-PK1 Cells"[MeSH Terms] OR "Madin Darby Canine Kidney Cells"[MeSH Terms] OR "MCF-7 Cells"[MeSH Terms] OR "NIH 3T3 Cells"[MeSH Terms] OR "PC12 Cells"[MeSH Terms] OR "PC-3 Cells"[MeSH Terms] OR "RAW 264.7 Cells"[MeSH Terms] OR "Sf9 Cells"[MeSH Terms] OR "Swiss 3T3 Cells"[MeSH Terms] OR "THP-

1 Cells"[MeSH Terms] OR "Tumor Cells, Cultured"[MeSH Terms] OR "Cells, Cultured"[MeSH Terms] OR "U937 Cells"[MeSH Terms] OR "Vero Cells"[MeSH Terms]) NOT "Computer Simulation"[MeSH Terms] NOT "Animals"[MeSH Terms:noexp] AND (hasabstract[text] AND English[lang])

"Human" (("Humans"[MeSH Terms:noexp] AND ("Organ Culture Techniques"[MeSH Terms] OR "Tissue Culture Techniques"[MeSH Terms] OR "Tissue Extracts"[MeSH Terms] OR "Actihaemyl"[MeSH Terms] OR "Cell Extracts"[MeSH Terms] OR "Liver Extracts"[MeSH Terms] OR "Pancreatic Extracts"[MeSH Terms] OR "Pancreatin"[MeSH Terms] OR "Pancrelipase"[MeSH Terms] OR "Placental Extracts"[MeSH Terms] OR "Thymus Extracts"[MeSH Terms] OR "Isolated Heart Preparation"[MeSH Terms] OR "Adult Germline Stem Cells"[MeSH Terms] OR "Adult Stem Cells"[MeSH Terms] OR "Blastomeres"[MeSH Terms] OR "Cellular Reprogramming Techniques"[MeSH Terms] OR "Embryoid Bodies"[MeSH Terms] OR "Embryonal Carcinoma Stem Cells"[MeSH Terms] OR "Embryonal Carcinoma Stem Cells"[MeSH Terms] OR "Embryonic Germ Cells"[MeSH Terms] OR "Embryonic Stem Cells"[MeSH Terms] OR "Fetal Stem Cells"[MeSH Terms] OR "Granulocyte-Macrophage Progenitor Cells"[MeSH Terms] OR "Hematopoietic Stem Cells"[MeSH Terms] OR "Human Embryonic Stem Cells"[MeSH Terms] OR "Induced Pluripotent Stem Cells"[MeSH Terms] OR "Lymphoid Progenitor Cells"[MeSH Terms] OR "Megakaryocyte-Erythroid Progenitor Cells"[MeSH Terms] OR "Mesenchymal Stem Cells"[MeSH Terms] OR "Mouse Embryonic Stem Cells"[MeSH Terms] OR "Multipotent Stem Cells"[MeSH Terms] OR "Myeloid Progenitor Cells"[MeSH Terms] OR "Myoblasts, Cardiac"[MeSH Terms] OR "Myoblasts, Skeletal"[MeSH Terms] OR "Myoblasts, Smooth Muscle"[MeSH Terms] OR "Myoblasts"[MeSH Terms] OR "Neoplastic Stem Cells"[MeSH Terms] OR "Neural Stem Cells"[MeSH Terms] OR "Oligodendrocyte Precursor Cells"[MeSH Terms] OR "Oogonial Stem Cells"[MeSH Terms] OR "Peripheral Blood Stem Cells"[MeSH Terms] OR "Pluripotent Stem Cells"[MeSH Terms] OR "Precursor Cells, B-Lymphoid"[MeSH Terms] OR "Precursor Cells, T-Lymphoid"[MeSH Terms] OR "Primary Cell Culture"[MeSH Terms] OR "Satellite Cells, Skeletal Muscle"[MeSH Terms] OR "Side-Population Cells"[MeSH Terms] OR "Stem Cell Research"[MeSH Terms] OR "Stem Cells"[MeSH Terms] OR "Thymocytes"[MeSH Terms] OR "Totipotent Stem Cells"[MeSH Terms] OR "3T3 Cells"[MeSH Terms] OR "A549 Cells"[MeSH Terms] OR "BALB 3T3 Cells"[MeSH Terms] OR "Caco-2 Cells"[MeSH Terms] OR "Cell Line, Transformed"[MeSH Terms] OR "Cell Line, Tumor"[MeSH Terms] OR "Cell Line"[All Fields] OR "CHO Cells"[MeSH Terms] OR "COS Cells"[MeSH Terms] OR "HCT116 Cells"[MeSH Terms] OR "HEK293 Cells"[MeSH Terms] OR "HeLa Cells"[MeSH Terms] OR "Hep G2 Cells"[MeSH Terms] OR "HL-60 Cells"[MeSH Terms] OR "HT29 Cells"[MeSH Terms] OR "Jurkat Cells"[MeSH Terms] OR "K562 Cells"[MeSH Terms] OR "L Cells (Cell Line)"[MeSH Terms] OR "LLC-PK1 Cells"[MeSH Terms] OR "Madin Darby Canine Kidney Cells"[MeSH Terms] OR "MCF-7 Cells"[MeSH Terms] OR "NIH 3T3 Cells"[MeSH Terms] OR "PC12 Cells"[MeSH Terms] OR "PC-3 Cells"[MeSH Terms] OR "RAW 264.7 Cells"[MeSH Terms]

OR "Sf9 Cells"[MeSH Terms] OR "Swiss 3T3 Cells"[MeSH Terms] OR "THP-1 Cells"[MeSH Terms] OR "Tumor Cells, Cultured"[MeSH Terms] OR "Cells, Cultured"[MeSH Terms] OR "U937 Cells"[MeSH Terms] OR "Vero Cells"[MeSH Terms] OR "Computer Simulation"[MeSH Terms])) AND "Diseases Category"[MeSH Terms]) NOT ("Clinical Study"[Publication Type] OR "Clinical Trial"[Publication Type] OR "Clinical Trial, Phase I"[Publication Type] OR "Clinical Trial, Phase II"[Publication Type] OR "Clinical Trial, Phase III"[Publication Type] OR "Clinical Trial, Phase IV"[Publication Type] OR "Clinical Trial, Veterinary"[Publication Type] OR "Clinical Trial Protocol"[Publication Type] OR "Controlled Clinical Trial"[Publication Type] OR "Clinical Conference"[Publication Type] OR "Review"[Publication Type]) NOT ("Animals, Newborn"[MeSH Terms] OR "Disease Models, Animal"[MeSH Terms] OR "Animals, Genetically Modified"[MeSH Terms] OR "Animal Experimentation"[MeSH Terms] OR "Vivisection"[MeSH Terms] OR "Models, Animal"[MeSH Terms] OR "Xenograft Model Antitumor Assays"[MeSH Terms] OR "Neoplasm Transplantation"[MeSH Terms] OR "Leukemia, Experimental"[MeSH Terms] OR "Liver Neoplasms, Experimental"[MeSH Terms] OR "Mammary Neoplasms, Experimental"[MeSH Terms] OR "Melanoma, Experimental"[MeSH Terms] OR "Sarcoma, Experimental"[MeSH Terms] OR "Encephalomyelitis, Autoimmune, Experimental"[MeSH Terms] OR "Myasthenia Gravis, Autoimmune, Experimental"[MeSH Terms] OR "Neuritis, Autoimmune, Experimental"[MeSH Terms] OR "Arthritis, Experimental"[MeSH Terms] OR "Diabetes Mellitus, Experimental"[MeSH Terms] OR "Liver Cirrhosis, Experimental"[MeSH Terms] OR "Neoplasms, Experimental"[MeSH Terms] OR "Nervous System Autoimmune Disease, Experimental"[MeSH Terms] OR "Radiation Injuries, Experimental"[MeSH Terms] OR "Mice"[MeSH Terms] OR "Mice, Knockout"[MeSH Terms] OR "Mice, Transgenic"[MeSH Terms] OR "Mice, Inbred C57BL"[MeSH Terms] OR "Mice, Nude"[MeSH Terms] OR "Mice, SCID"[MeSH Terms] OR "Mice, Congenic"[MeSH Terms] OR "Mice, Inbred Strains"[MeSH Terms] OR "Mice, Mutant Strains"[MeSH Terms] OR "Mice, Obese"[MeSH Terms] OR "Mice, 129 Strain"[MeSH Terms] OR "Mice, Hairless"[MeSH Terms] OR "Rats"[MeSH Terms] OR "Rats, Sprague-Dawley"[MeSH Terms] OR "Rats, Wistar"[MeSH Terms] OR "Rats, Transgenic"[MeSH Terms] OR "Rats, Mutant Strains"[MeSH Terms] OR "Rats, Long-Evans"[MeSH Terms] OR "Rats, Inbred Strains"[MeSH Terms]) NOT "Animals"[MeSH Terms:noexp] AND (hasabstract[text] AND English[lang])

"Invertebrates" "Drosophila" OR "Drosophila melanogaster" OR "Caenorhabditis" OR "Caenorhabditis elegans" OR "Caenorhabditis/analysis" OR "Caenorhabditis/anatomy and histology" OR "Caenorhabditis/chemistry" OR "Caenorhabditis/classification" OR "Caenorhabditis/cytology" OR "Caenorhabditis/drug effects" OR "Caenorhabditis/embryology" OR "Caenorhabditis/enzymology" OR "Caenorhabditis/etiology" OR "Caenorhabditis/genetics" OR "Caenorhabditis/growth and development" OR "Caenorhabditis/immunology" OR "Caenorhabditis/isolation and purification" OR "Caenorhabditis/metabolism" OR "Caenorhabditis/microbiology" OR "Caenorhabditis/parasitology" OR "Caenorhabditis/pathogenicity"

OR "Caenorhabditis/physiology" OR "Caenorhabditis/radiation effects" OR "Caenorhabditis/ultrastructure" OR "Caenorhabditis/virology" OR "Caenorhabditis elegans/analysis" OR "Caenorhabditis elegans/anatomy and histology" OR "Caenorhabditis elegans/chemistry" OR "Caenorhabditis elegans/classification" OR "Caenorhabditis elegans/cytology" OR "Caenorhabditis elegans/drug effects" OR "Caenorhabditis elegans/embryology" OR "Caenorhabditis elegans/enzymology" OR "Caenorhabditis elegans/etiology" OR "Caenorhabditis elegans/genetics" OR "Caenorhabditis elegans/growth and development" OR "Caenorhabditis elegans/immunology" OR "Caenorhabditis elegans/isolation and purification" OR "Caenorhabditis elegans/metabolism" OR "Caenorhabditis elegans/microbiology" OR "Caenorhabditis elegans/parasitology" OR "Caenorhabditis elegans/pathogenicity" OR "Caenorhabditis elegans/physiology" OR "Caenorhabditis elegans/radiation effects" OR "Caenorhabditis elegans/ultrastructure" OR "Caenorhabditis elegans/virology" OR "Drosophila/analysis" OR "Drosophila/anatomy and histology" OR "Drosophila/chemistry" OR "Drosophila/classification" OR "Drosophila/cytology" OR "Drosophila/diagnosis" OR "Drosophila/diagnostic imaging" OR "Drosophila/drug effects" OR "Drosophila/embryology" OR "Drosophila/enzymology" OR "Drosophila/epidemiology" OR "Drosophila/ethnology" OR "Drosophila/etiology" OR "Drosophila/genetics" OR "Drosophila/growth and development" OR "Drosophila/immunology" OR "Drosophila/isolation and purification" OR "Drosophila/metabolism" OR "Drosophila/microbiology" OR "Drosophila/parasitology" OR "Drosophila/pathogenicity" OR "Drosophila/pharmacology" OR "Drosophila/physiology" OR "Drosophila/radiation effects" OR "Drosophila/statistics and numerical data" OR "Drosophila/ultrastructure" OR "Drosophila/virology" OR "Drosophila melanogaster/analysis" OR "Drosophila melanogaster/anatomy and histology" OR "Drosophila melanogaster/chemistry" OR "Drosophila melanogaster/classification" OR "Drosophila melanogaster/cytology" OR "Drosophila melanogaster/drug effects" OR "Drosophila melanogaster/embryology" OR "Drosophila melanogaster/enzymology" OR "Drosophila melanogaster/etiology" OR "Drosophila melanogaster/genetics" OR "Drosophila melanogaster/growth and development" OR "Drosophila melanogaster/immunology" OR "Drosophila melanogaster/isolation and purification" OR "Drosophila melanogaster/metabolism" OR "Drosophila melanogaster/microbiology" OR "Drosophila melanogaster/parasitology" OR "Drosophila melanogaster/pathogenicity" OR "Drosophila melanogaster/physiology" OR "Drosophila melanogaster/radiation effects" OR "Drosophila melanogaster/ultrastructure" OR "Drosophila melanogaster/virology"

2 Annotation guidelines

The GoldHamster II corpus is set up to enable the training of machine learning algorithms for the classification of PubMed abstracts regarding the applied method (e.g. research with primary cells).

The aim of the annotation is to identify and to highlight the type of applied method on the basis of PubMed abstracts. The applied methods shall be categorized according to eight labels listed below:

- In vivo experiments in living vertebrates and cephalopods (in_vivo)
- Vertebrate organs and tissues (organs)
- Vertebrate primary and stem cells (primary_cells)
- Vertebrate immortalized and cancer cell lines (immortal_cell_line)
- Experiments with humans or human material (human)
- Experiments with invertebrates or invertebrate material (excluding cephalopods) (invertebrate)
- In silico (in_silico)
- Others (other)

The respective type of method is named in parenthesis as configured in the tool that we use for annotation (called TeamTat[1]). There are general guidelines for the annotation of the documents:

- The annotation will be carried out by means of highlighting the text with the support of the tool.
- Please highlight only the applied method most likely used in the respective study. Please do not annotate (highlight) methods (models) cited in the text in another context, e.g. as background information in the introduction or discussion sections of the abstract. Some articles also cite species in other context, such as in the example below, which describes an in silico (BLAST) method:

METHODS: In this study we used the BLAST reciprocal best-hit methodology to search for DDR orthologs proteins in Aedes aegypti. We also provided a comparison between Ae. aegypti, D. melanogaster and human DDR network. (PMID 31711518)

- The annotation shall be carried out based on the title and abstract. Indeed, we noticed that in some articles, the proposed method is only cited in the title but not in the abstract.

[1] `https://www.teamtat.org/`

- The identified applied method should be annotated and highlighted according to one of the eight labels.
- It is possible to annotate and highlight two or more methods (e.g. if an in vivo experiment in mice and an experiment with immortalized cell lines is conducted). It is necessary to highlight at least one text span for each label, but it is not necessary to annotate all mentions of the same method.
- However, feel free to annotate all mentions of the same methods, if possible, since this is valuable information for us or for the machine learning. These mentions can provide interesting information regarding the location of the proposed mention in the text or its various forms, e.g., "nematode", "Caenorhabditis elegans", "C. elegans" and "worms" (all present in PMID 31713185).
- The length of the highlighted text is not relevant, in case of multi-words. For instance, either "pig coronary arteries in vitro" or "pig coronary arteries" are valid annotations, as long as assigned to the correct label (in this case "organs").
- To annotate the labels and highlight the methods correctly, you are allowed to use Google or any other search strategy, if, for example, abbreviations or names of cell lines and other methods are unknown.
- If the document does not contain an abstract, it should be ignored, i.e., no annotation at all should be carried out.
- If no label can be allocated, the document should be ignored, i.e., no annotation at all should be carried out. However, the status of the document should be set to "annotated" (cf. "Done" button in Figure 5).

For each label, we provide a short description, explain what the annotator should highlight in the text and show one example from the corpus.

2.1 In vivo experiments on vertebrates, cephalopods

Experiments using certain living animals, i.e. vertebrates like mice, rats, pigs, zebrafish, or cephalopods like octopusses. Those animals are protected by the German Animal Protection Act.

What to annotate? Please highlight the described use of living animals.

Study was carried out on rats, divided into four groups; negative control, positive control, standard drug control, and Myristica fragrans extract treated rats. (PMID 31241706)

2.2 Vertebrate organs and tissues

Experiments using organs or tissues of vertebrate animals that were killed in advance.

What to annotate? Please highlight the described organ- or tissue-based method.

This study describes the effect of variable oxygen supply on relaxing responses induced by alpha-calcitonin gene-related peptide (CGRP) and adrenomedullin (AM) on isolated pig coronary arteries in vitro. (PMID 24084221)

2.3 Vertebrate primary and stem cells

Experiments using primary cells or stem cells that were obtained from vertebrate animals. See `https://web.expasy.org/cgi-bin/cellosaurus/search` "finite" or "stem" for examples.

What to annotate? Please highlight the described primary or stem cell method.

Rabbit tracheal ciliated cell culture was established and CBF was determined using high-speed digital imaging methods. (PMID 22508392)

2.4 Vertebrate immortalized (transformed) and cancer cell lines

Experiments using immortal (transformed) and cancer cell lines (from vertebrate animals) that may be obtained from a provider like ATCC (American Type Culture Collection). See `https://web.expasy.org/cgi-bin/cellosaurus/search` "immortal" for examples.

What to annotate? Please highlight the described immortal cell line method.

This study was conducted to investigate the susceptibility of the porcine ileum epithelial cell line, IPI-2I, to different swine enteric CoVs. (PMID 31176408 and Cellosaurus[2])

2.5 Experiments with humans or human materials

Experimental research using living humans or human organs, tissues, stem cells, primary cells, immortalized or cancer cells.

[2] `https://web.expasy.org/cellosaurus/CVCL_3826`

What to annotate? Please highlight the described use of humans or human material.

On this account, our study was directed to scrutinize the influence of this triterpenoid on human hepatocellular cancer cell model Hep3B. (PMID 31250646)

2.6 Experiments with invertebrates or invertebrate materials

Experiments using specific animal species, i.e. invertebrates like Drosophila, Caenorhabditis (C.) elegans. Those animals are NOT protected by the Directive 2010/63/EU.

What to annotate? Please highlight the described (invertebrate) animal species.

Drosophila CRYPTOCHROME (dCRY) mediates electrophysiological depolarization and circadian clock resetting in response to blue or ultraviolet (UV) light. [. . .] To examine these issues in in vivo and in ex vivo whole-brain preparations, we generated transgenic flies expressing tryptophan mutant dCRYs in the conserved electron transfer chain and then measured neuronal electrophysiological phototransduction and behavioral responses to light. (PMID 31659046)

2.7 In silico

Experiments with virtual models.

What to annotate? Please highlight the described in silico model.

A computational model equipped with the main immunological features of the sea bass (Dicentrarchus labrax L.) immune system was used to predict more effective vaccination in fish. (PMID 28549079)

2.8 Other

This includes methods and approaches not covered by any of the above labels, such as human observational studies, in chemico research or use of larvae or early embryos (see attached figure)

What to annotate? Please highlight the described method.

OBJECTIVE: To determine any changes in total hospital revisits within 30 days

of discharge after a hospital stay for medical conditions targeted by the Hospital Readmissions Reduction Program (HRRP). DESIGN: Retrospective cohort study. SETTING: Hospital stays among Medicare patients for heart failure, acute myocardial infarction, or pneumonia between 1 January 2012 and 1 October 2015. (PMID 31405902)

3 Analysis of the MeSH terms

We computed the variation on the number of MeSH terms per document according to a range of values for the threshold. This is depicted in the figure below.

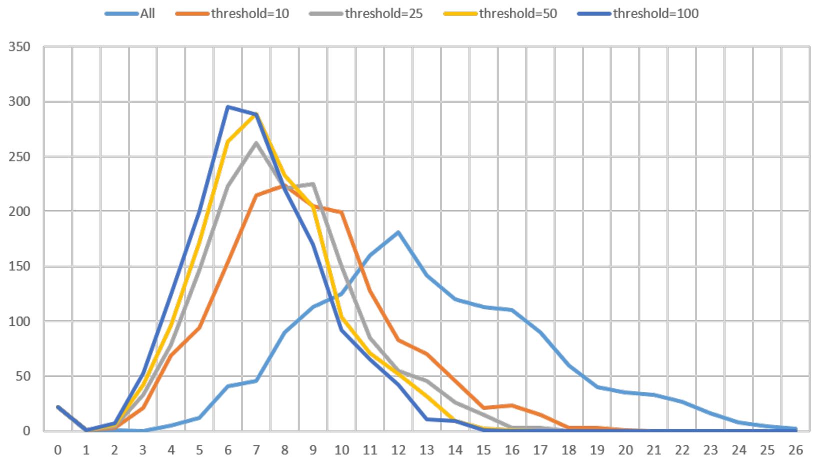

Figure 1: The vertical axis corresponds to the number of documents, and the horizontal axis is the number of Mesh terms per document.

4 Experiments with section labels

For obtaining the sections from the structured abstracts, we first checked the "NlmCategory" attribute of the abstract text element, and if not found, we considered the "Label" attribute of this element. The final list of sections that we considered were the following (along with the number of times they occurred): RESULTS (423), CONCLUSIONS (405), BACKGROUND (226), METHODS (364), OBJECTIVE (221), CONCLUSION (22), Results (17), Methods (12), Background (11), and Conclusion (11). This set of sections covers 454 abstracts that contain at least one of the above sections. Details about the mapping between the section of ArguminSci and the Structured Abstracts from PubMed are shown in Table 1 below.

Table 1: Mapping to the original section names.

Labels	Structured abstracts	ArguminSci
Background	BACKGROUND, Background	DRI_Background
Objective	OBJECTIVE	DRI_Challenge
Methods	METHODS, Methods	DRI_Approach
Results	RESULTS, Results	DRI_Outcome
Conclusions	CONCLUSIONS, CONCLUSION, Conclusion	DRI_FutureWork

From the ArguminSci tool, we obtained five labels (along with the number of times they occurred in our collection of 1,600 abstracts): DRI_Background (1565), DRI_Challenge (858), DRI_Approach (1348), DRI_Outcome (1373), DRI_FutureWork (27).

5 PMIDs removed in the second rounds

The following PMIDs were removed from the corpus during the additional rounds of annotations.

- r2.1: 31708395, 31659041, 30638522, 30551685, 31697328, 30658692, 31661072, 28237890, 26108956, 22216973, 27870267

- r2.2: 31405409, 31366381, 31181314, 31212245, 27600312, 31116244, 31357966, 31366540, 31374004, 31366539, 31405426, 31147664, 31405410, 31375236, 31366513, 31366563, 31374005, 31251971, 31330234, 30502910

- r2.3: 29065140, 30580951, 31029149, 26528898, 29728336, 31170779, 24478394, 31171772, 20197146, 30131013, 29304962, 27139488, 31171792, 27246553, 29730007, 30468255, 30473344, 27833150, 31046828, 28582594, 30744668, 29466325, 30808820

- r2.4: 20581727, 27167122, 27432751, 27557522, 27576237, 27642364, 27889557, 27907920, 28116677, 28215146, 28237916, 28652198, 28684049, 28763035, 28861630, 28894166, 30136079, 30261281, 30400299, 30423344, 30477957, 30488967, 30499009, 30511462, 30529563, 30557577, 30572344, 30583231, 30598996, 30631959, 30658691, 30683754, 30696089, 30706195, 30707600, 30717094, 30827382, 30893481, 30903378, 30930221, 30949853, 31138793, 31138806, 31140843, 31175300, 31186408, 31188028, 31200252, 31347933, 31387681, 31387684, 31392878, 31654871, 31674725, 31698692, 31698957, 31699221, 31366549, 31227715, 31084896, 28412844, 31145345, 31375147, 30556178, 31222023, 31262901, 21318873, 31393863, 30488503, 30993463, 31073706, 26865366, 31221064, 25451973, 23509978, 29397416, 31209400

2

6 Statistics of the corpus

Statistics of the training, development and test sets for the corpus after all rounds of annotations.

Table 2: Statistics of the corpus (average values for the 10-fold cross validation).

	train	dev	test
none	22.5	1.3	1.2
human	201.6	11.2	11.2
invertebrate	190.8	10.4	10.8
in_vivo	368.1	20.5	20.4
other	210.6	11.5	11.9
primary_cells	84.6	4.9	4.5
immortal_cell_line	93.6	5.3	5.1
organs	144.9	7.8	8.3
in_silico	141.3	8.1	7.6

7 Selection of the hyperparameters

We carried out some experiments to decide the best set of hyperparameters, for which we tried the following values:

- batch size: 16 and 32;
- learning rate: 1×10^{-5}, 5×10^{-5}, 1×10^{-4}, 5×10^{-4};
- epochs: 10, 20, 30, 40, 50.

All experiments were carried out for the following configuration:

- fold 3 of the cross-validation;
- annotations from rounds 1, 2.1, and 2.2;
- only annotations with agreement;
- the two cell line labels merged into one label.

We present three graphs of the results that we obtained, in which we highlight the distinct values for one of the hyperparameters above. For all figures, the value of the f-score which is depicted is the average one across all labels.

In Figure 2, the results for the highest learning rate (5×10^{-4}) were the lowest ones, while top results were obtained for (1×10^{-4}). Figure 3 shows that there was little impact of the batch size in the results, since both values (16 or 32) obtained low and high scores. Finally, Figure 4 present high and low results for all epochs, but the best result was obtained with 20 epochs.

Based on the the above experiments, the highest F-score of 0,8225 was obtained when considering only the following hyperparameters: batch size of 16, learning rate of 1×10^{-4}, and 20 epochs.

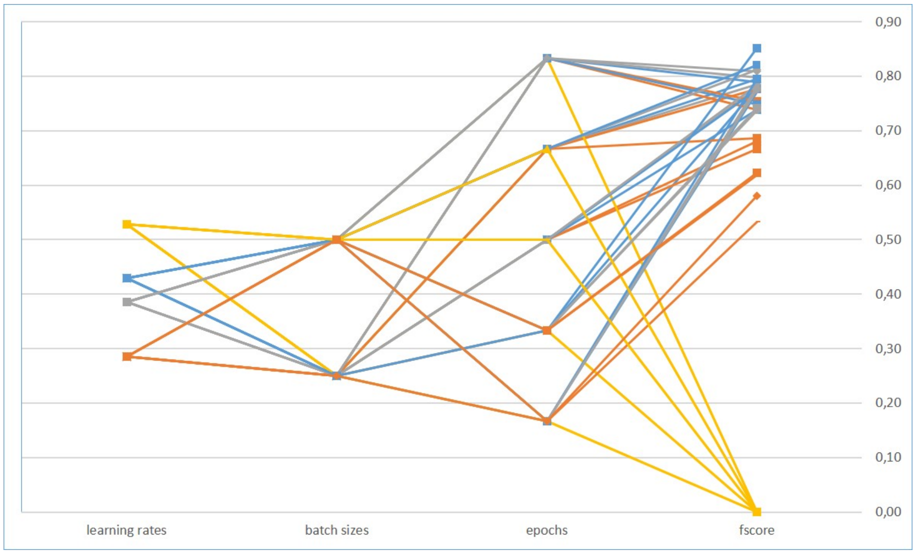

Figure 2: Hyperparameters with focus on the learning rates. The F-score is shown on the right. The values for the three hyperparameters are the following (from bottom to top): (a) learning rates: 1×10^{-5} (bottom), 5×10^{-5}, 1×10^{-4}, 5×10^{-4} (top); (b) batch sizes: 16 (bottom) and 32 (top); and (c) epochs: 10 (bottom), 20, 30, 40, and 50 (top).

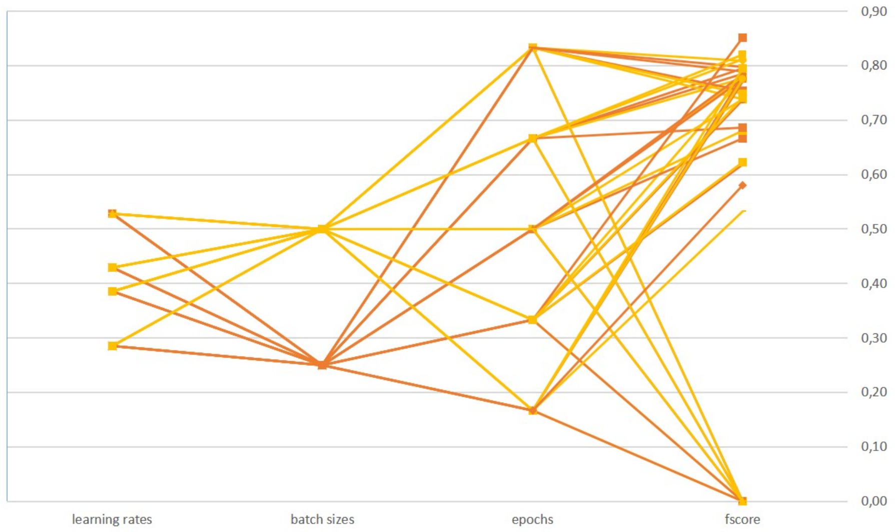

Figure 3: Hyperparameters with focus on the batch sizes. The F-score is shown on the right. The values for the three hyperparameters are the following (from bottom to top): (a) learning rates: 1×10^{-5} (bottom), 5×10^{-5}, 1×10^{-4}, 5×10^{-4} (top); (b) batch sizes: 16 (bottom) and 32 (top); and (c) epochs: 10 (bottom), 20, 30, 40, and 50 (top).

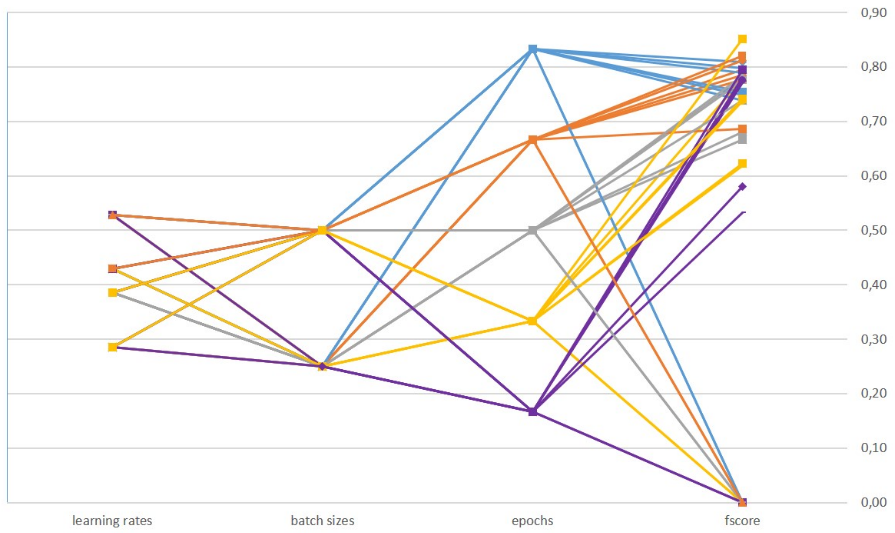

Figure 4: Hyperparameters with focus on the epochs. The F-score is shown on the right. The values for the three hyperparameters are the following (from bottom to top): (a) learning rates: 1×10^{-5} (bottom), 5×10^{-5}, 1×10^{-4}, 5×10^{-4} (top); (b) batch sizes: 16 (bottom) and 32 (top); and (c) epochs: 10 (bottom), 20, 30, 40, and 50 (top).

8 Results for Support Vector Machines

We considered the SVM classifier as implemented in the Python scikit-learn library[3]. We tried the four kernel functions[4] available in the library, namely linear, polynomial, RBF, and sigmoid. All experiments utilized a TF-IDF representation of the abstracts and we ran various experiments in order to achieve the best parameters for each of the kernel functions.

We ran experiments for the four kernel functions, i.e. linear, polynomial, radial basis function (RBF), and sigmoid We also experimented with a set of values for the hyperparameters which are required by each kernel function, as shown below:

- RBF: “gamma” of 0.25, 0.5, 0.6, 0.75, 0.8, 1 and 2
- sigmoid: “coef0” of -1, -0.5, -0.25, 0, 0.25, 0.5 and 1
- polynomial: “degree” of 2, 3 and 4; “coef0” of -100, -50, -10, -1, 0, 1, 10, 50, 100

The best performances obtained by each kernel function are summarized in Table 3 and the best hyperparameters were the following: “gamma” of 0.60 for RBF, “coef0” of -0.25 for sigmoid, and “degree” of 2 and “coef0” of 100 for polynomial.

Table 3: Performance of the methods for the prediction of the labels. We show the average of the f-scores that we obtained for each method in a 10-fold cross-validation.

Labels	SVM				PubMedBERT
	linear	RBF	sigmoid	polynomial	
invertebrates	0.82	0.52	0.82	0.83	0.95
in_vivo	0.73	0.58	0.73	0.76	0.83
human	0.56	0.43	0.56	0.62	0.73
organs	0.48	0.08	0.47	0.61	0.71
primary_cells	0.07	0.00	0.07	0.26	0.67
immortal_cell_line	0.21	0.00	0.18	0.51	0.89
in_silico	0.59	0.17	0.56	0.64	0.86
other	0.46	0.29	0.44	0.49	0.76
All (average)	0.38	0.14	0.37	0.53	0.83

[3]`https://scikit-learn.org/stable/modules/svm.html`
[4]`https://scikit-learn.org/stable/modules/svm.html#svm-kernels`

9 Evaluation of NER tools

We also evaluated the performance of the NER tools when considering only the annotations that occur in some particular discourse elements. Table 4 summarizes the results.

Table 4: Performance (in f-score) when only considering annotations from particular discourse elements. We show results from considering each section separately, i.e. Background (B), Objective (O), Methods (M), Results (R), and Conclusions (C).

**BERN2**	**none**	**B**	**O**	**M**	**R**	**C**
invertebrates	0.54	0.30	0.03	0.26	0.16	0.00
in_vivo	0.56	**0.57**	0.31	0.39	0.44	0.01
human	0.52	0.52	0.22	0.33	0.31	0.02
immortal_cell_line	0.24	**0.27**	0.15	0.26	0.30	0.00
**PubTator**	**none**	**B**	**O**	**M**	**R**	**C**
invertebrates	0.72	0.44	0.13	0.40	0.33	0.00
in_vivo	0.61	0.59	0.30	0.41	0.46	0.01
human	0.45	**0.46**	0.21	0.29	0.27	0.02
immortal_cell_line	0.00	0.00	0.00	0.00	0.00	0.00

10 Annotations - error analysis

We list the most frequent labels with disagreements in Tabel 5 below. Of the 20 disagreements listed in this table, 15 of them concern cases where one of the annotators assigned either no label, or the label "others". This high proportion possibly indicates that annotators might have chosen to assign the "others" label, or no label at all, when encountering an abstract where labeling was unclear. We analyzed each of the disagreements (cf. table) and we summarize below the possible reasons for the misunderstandings:

- Misunderstanding of the annotation guideline, which stipulates to label only according to the actually used experimental model as described in the methodical part of the abstract, but not according to parts of the abstract that constitute related work or background information. [not-used-method]
- Mistake regarding the origin of cell lines. The guideline requires to label cell lines according to source organism: cells from invertebrates as "invertebrates", while the ones derived from humans as "human" [cell-line-origin]
- Difficulty in identifying whether an animal was killed in advance and its organs or tissues were used subsequently in experiments (label "organs"), or whether the living animal was used for experiments (label "in vivo"). Furthermore, situations in which the authors cite a certain animal organ or tissue in another context, but they did not refer to an ex vivo experiment. [organs-or-invivo]
- Inability to distinguish whether an experiment that involves an animal was indeed an animal experiment according to the EU guidelines. [animal-experim]
- Difficulty in deciding in cases where none of the labels fits an abstract, i.e., whether to assign any label at all, or to assign the label "others" instead. [none-or-others]
- Problem in deciding whether the experimental model described in an abstract corresponds to one of the labels or whether the abstract should be assigned to the "others" label instead. [label-or-others]
- Complexity of the abstract or lack of enough details about the used model. These are cases in which the abstract describes several models and methods without giving enough details, and thus making the identification of a relevant label difficult. [unclear-method]

Table 5: Number of the articles (No.) without full agreement according to their labels and their combinations. We only show the disagreements that occurred at least 10 times. Details about the possible reasons of disagreement were described in details in the text.

No.	ann1	ann2	reason	PMID	disagreement
55	human	others	label-or-others	31357966	case report
46	invivo	invivo, organs	organs-or-invivo	31108095	Hematoxylin-eosin
32	-	others	none-or-others	30151648	review paper
26	human	imm._c.	cell-line-origin	31147664	ovarian cancer cells
22	imm._c.	prim._c.	cell-line-origin	30670030	TMUV-infect. BHK-21
20	invivo	invivo+prim._c.	animal-experim	30556415	embryos
20	human	-	unclear-method	31221068	patient-specific iPSCs
20	human	human+others	label-or-others	31405409	retrospective study
19	others	human+others	label-or-others	31393364	meta-analysis
19	invert.	others	label-or-others	31666534	Drosophila larval
19	invivo	-	unclear-method	22705061	rabbits
18	invivo	others	animal-experim	27246553	dogs
16	insilico	others	label-or-others	29397416	G. hirsutum plants
14	invert.	-	not-used-meth	31661072	Drosophila
13	invivo	organs	organs-or-invivo	30355727	zebrafish heart
12	invivo	invivo+others	none-or-others	31170996	recomb. B. subtilis
12	insilico	-	label-or-others	28237916	parametric study
11	insilico	insilico+others	none-or-others	29032659	A. pleuropneumoniae
10	-	prim._c.	not-used-method	31221064	Mesench. stromal cells
10	invert.	invert.+others	none-or-others	31706086	S. marcescens
